# Supplementary material for: A threonyl-tRNA synthetase-mediated translation initiation machinery
Source: Nat Commun. 2019 Mar 22;10:1357. doi: 10.1038/s41467-019-09086-0 (PMC6430810; doi:10.1038/s41467-019-09086-0)
Supplement: Supplementary file 1 — Supplementary Information [file 41467_2019_9086_MOESM1_ESM.pdf]

Supplementary Information

A threonyl-tRNA synthetase-mediated translation initiation machinery

Jeong *et al*.

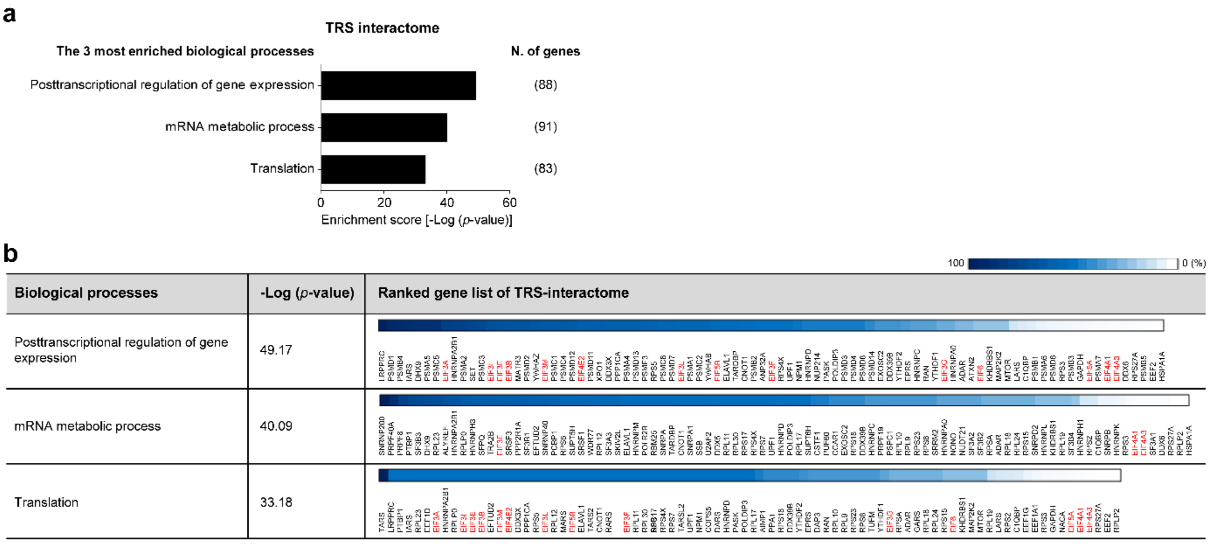

c

Gene symbol	Description	Entrez gene
TARS	Threonyl-tRNA synthetase	6897
EIF4E2	Eukaryotic translation initiation factor 4E family member 2	9470
TXN	Thioredoxin	7295
GINS2	GINS complex subunit 2	51659

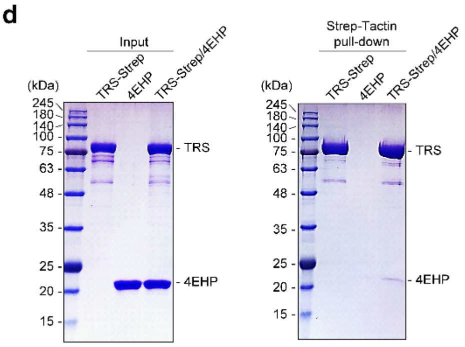

Supplementary Figure 1 TRS is involved in translation by interacting with eukaryotic translation initiation factors. **a** GO enrichment analysis of the TRS interactome. The number of genes associated with each GO term is shown in the right column. The three most enriched GO terms in the Biological Process category are shown. **b** Ranked gene list associated with TRS in the indicated biological processes. Genes encoding eukaryotic translation initiation factors are highlighted in red. **c** Candidate proteins interacting with TRS based on yeast two-hybrid data. **d** *In vitro* pull-down assay of purified full-length TRS-Strep and 4EHP. TRS-Strep was pulled down with Strep-Tactin beads, and co-precipitated 4EHP was eluted from the resin. The eluted samples were separated on SDS-PAGE and visualized by Coomassie staining. Data are representative of at least three experiments, each with similar results (**d**).

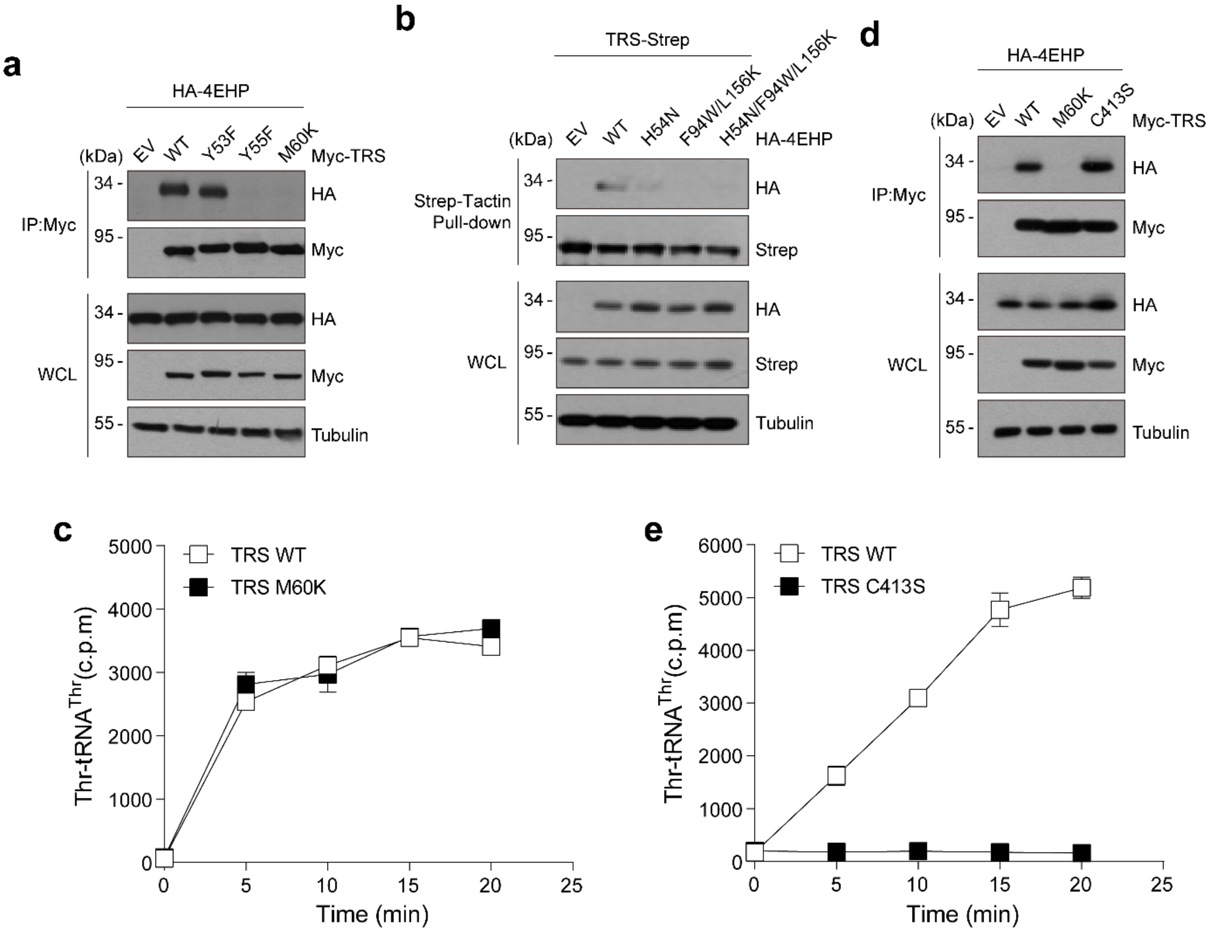

Supplementary Figure 2 Critical residues involved in the TRS and 4EHP interaction. **a** Immunoassay of co-expressed WT or mutant Myc-TRS with HA-4EHP in 293T cells. Myc-TRS was immunoprecipitated with anti-Myc antibody, and co-precipitated 4EHP was detected by immunoblotting with anti-HA antibody. EV, empty vector; WCL, whole cell lysate. **b** Pull-down assay of co-expressed WT or mutant HA-4EHP with TRS-Strep in 293T cells. TRS-Strep was pulled down with Strep-Tactin beads, and co-precipitated 4EHP was detected by immunoblotting with anti-HA antibody. **c** Catalytic activities of WT TRS and the 4EHP-binding defective M60K mutant determined by threonylation of $tRNA^{Thr}$ as described in the Methods. The amount of threonine-charged tRNA was measured by scintillation counting, and data (mean ± SD of three independent experiments) are displayed as line graphs. c.p.m., counts per min. **d** Immunoassay of co-expressed HA-4EHP with WT Myc-TRS, the 4EHP-binding

defective M60K mutant, or the catalytically defective C413S mutant in 293T cells. TRS was immunoprecipitated with anti-Myc antibody, and co-precipitation of HA-4EHP was detected by immunoblotting with anti-HA antibody. **e** Catalytic activities of WT TRS and the catalytically inactive C413S mutant determined as described in **c**. Values are means ± SD of three independent experiments.

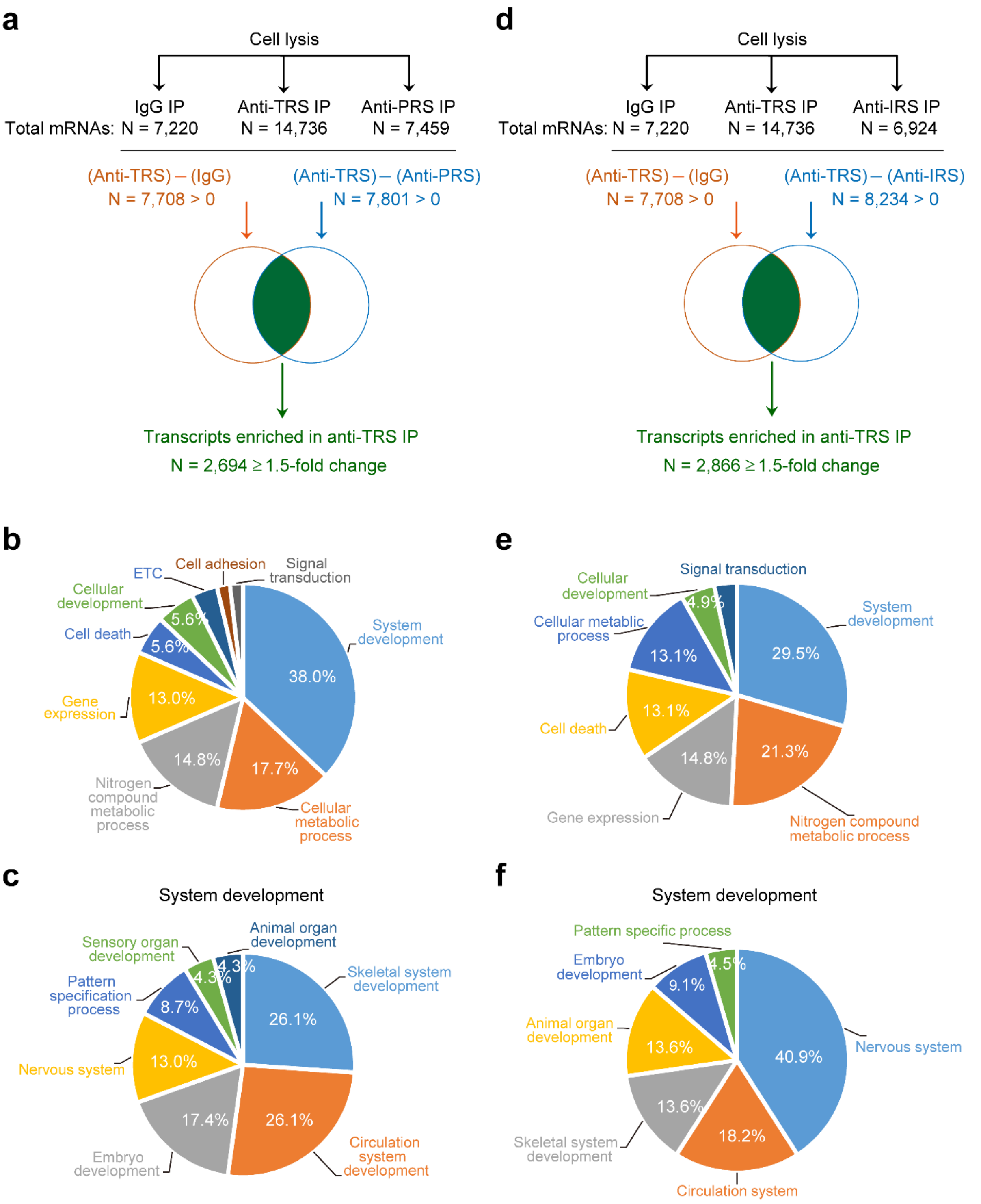

Supplementary Figure 3 TRS-mediated translation initiation control of mRNAs in vertebrates. **a** Workflow used to identify TRS-targeted mRNAs. Total mRNAs isolated from 293T cells were precipitated with anti-TRS or -PRS antibodies and/or mock IgG, and RNA sequencing of precipitated transcripts was conducted. TRS-enriched RNAs

were subtracted from those enriched in PRS and IgG groups, and the two RNA pools were compared to identify common transcripts. Among the transcripts commonly detected in both TRS-enriched pools, 2,694 ≥1.5-fold were selected, and subsequently subjected to functional annotation. **b**, **c** Functional annotation of TRS-targeted mRNAs. Enriched GO terms in the Biological Process category were analyzed using the Database for Annotation, Visualization and Integrated Discovery (DAVID). **d** Workflow used to identify TRS-targeted mRNAs. Total mRNAs isolated from 293T cells were precipitated with anti-TRS or -IRS antibodies and/or mock IgG, and RNA sequencing of precipitated transcripts was conducted. TRS-enriched RNAs were subtracted from those enriched in IRS and IgG groups, and the two RNA pools were compared to identify common transcripts. Among the transcripts commonly detected in both TRS-enriched pools, 2,866≥1.5-fold were selected, and subsequently subjected to functional annotation. **e**, **f** Functional annotation analyzed as described in **b**, **c**.

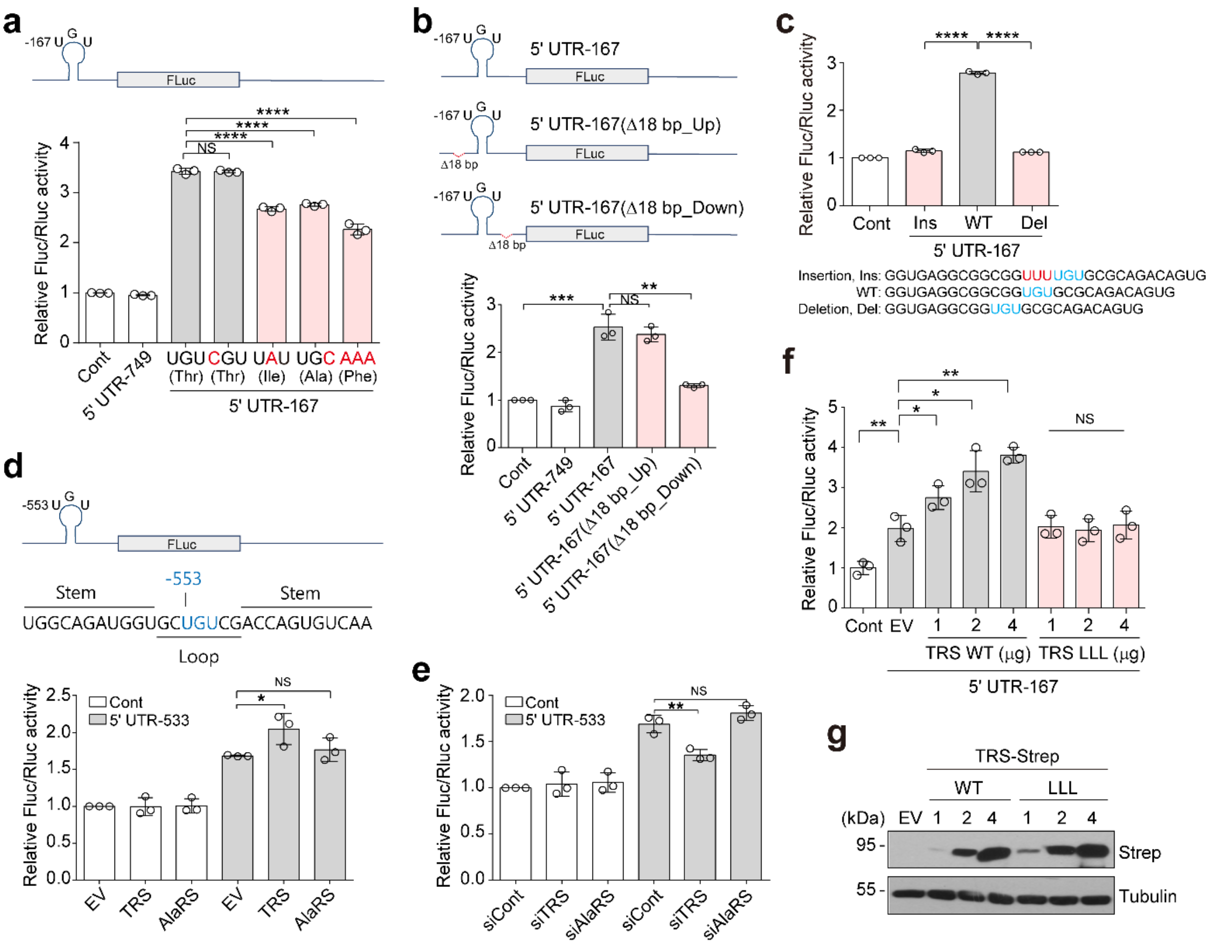

Supplementary Figure 4 TRS selects target mRNAs. **a** Effects of mutations at the pseudo-anticodon (-167 site) of *VEGF* mRNA on translation of the reporter gene in TRS-expressing 293T cells. Mutated sites are indicated in red, and the corresponding amino acid of each mutated anticodon is labeled in parentheses. **b** Effects of the pseudo-anticodon loop position from the translation start site on the translation of the reporter gene in TRS-expressing 293T cells. The loop position was changed by the deletion of 18 bp in upstream or downstream of the loop. Deleted sites are indicated by the red dashed line. Δ18 bp_Up, 18 bp deletion in the upstream of the loop; Δ18 bp_Down, 18 bp deletion in the downstream of the loop. **c** Effects of loop length on translation of the reporter gene in TRS-expressing 293T cells. The loop length was increased or reduced by the insertion (Ins) or deletion (Del) of bases into the pseudo-

anticodon UGU in the 5′ UTR-167. Inserted bases are shown in red. **d** Potential $tRNA^{Thr}$ anticodon triplet (UGU)-containing stem-loop structures were observed at positions -553 upstream from the *ANG* mRNA initiation codon. The RNA sequences spanning positions -19 to -600 (5′ UTR-553) were fused upstream of *Fluc* and co-expressed with *Rluc* in TRS- or AlaRS-expressing 293T cells. **e** Translation of the pseudo-anticodon-containing reporter gene in siTRS- or siAlaRS-transfected 293T cells. **f** Effects of the TRS anticodon-binding residues on translation of the reporter gene. The dual luciferase assay was performed on 293T cells expressing WT TRS-Strep and the RER triple mutant (R663L/E680L/R689L) TRS-Strep at 1–4 μg. The pseudo-anticodon-containing reporter gene was co-expressed with *Renilla* luciferase to monitor TRS-dependent and nonspecific translation, respectively (**a**–**f**). Data are presented as the ratio of *firefly* to *Renilla* luciferase activity (Fluc/Rluc). EV, empty vector; siCont, non-targeting control siRNA. LLL, R663L/E680L/R689L mutant TRS. *, $p < 0.05$; **, $p < 0.01$; ****, $p < 0.0001$; NS, not significant vs. control group. Values are means ± SD of three independent experiments. **g** Immunoblot analysis of WT TRS-Strep and LLL mutant TRS-Strep expressed in 293T cells using Strep antibody. Data are representative of at least three experiments, each with similar results.

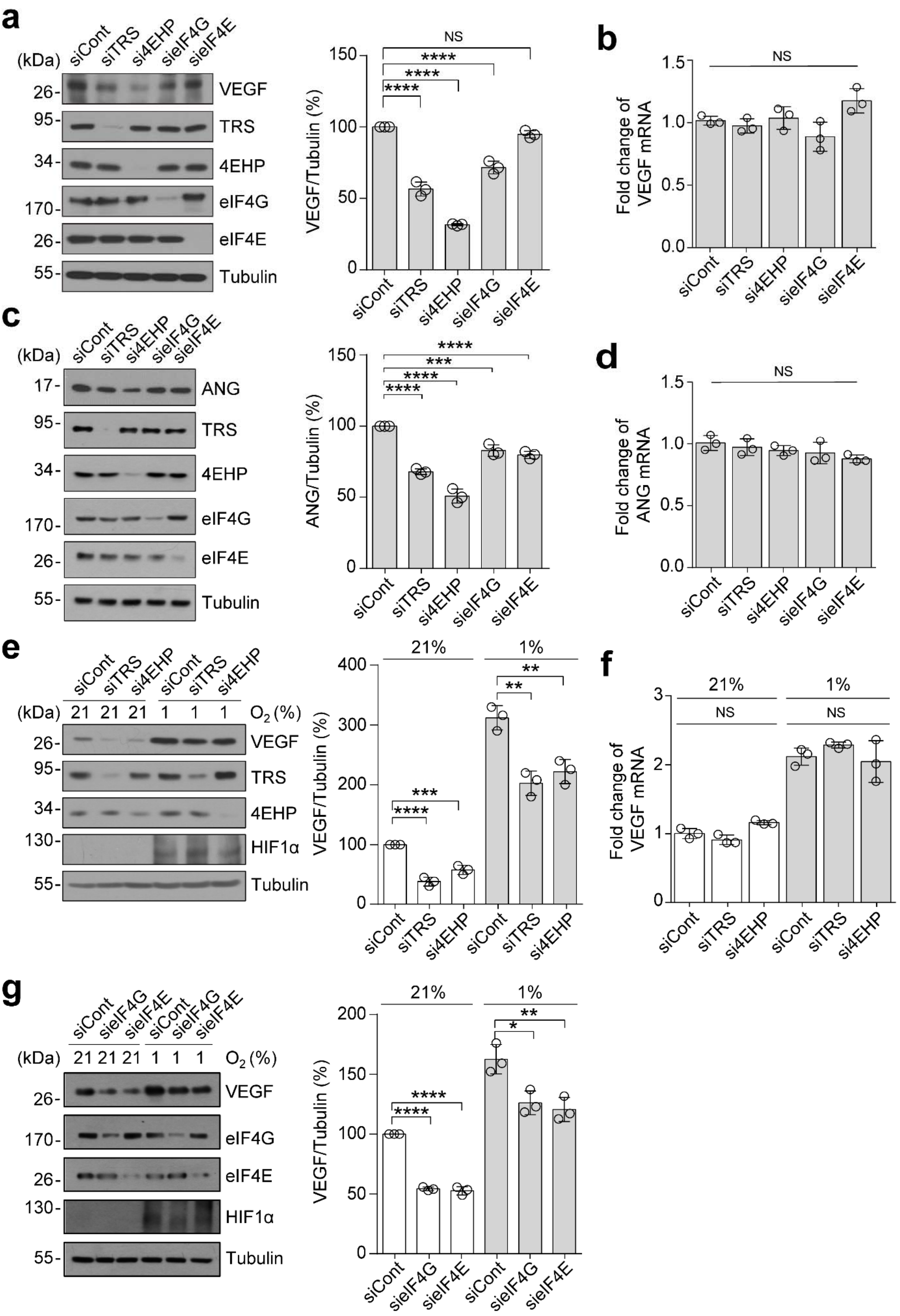
a
(kDa)
siCont
siTRS
si4EHP
sieIF4G
sieIF4E
26-
VEGF
95-
TRS
34-
4EHP
170-
eIF4G
26-
eIF4E
55-
Tubulin
VEGF/Tubulin (%)
NS
****
****
****
150
100
50
0
b
Fold change of VEGF mRNA
NS
1.5
1.0
0.5
0.0
c
(kDa)
17-
ANG
95-
TRS
34-
4EHP
170-
eIF4G
26-
eIF4E
55-
Tubulin
ANG/Tubulin (%)
****
***
****
****
d
Fold change of ANG mRNA
NS
e
O2 (%)
21 21 21 1 1 1
26-
VEGF
95-
TRS
34-
4EHP
130-
HIF1α
55-
Tubulin
VEGF/Tubulin (%)
21%
1%
***
****
**
**
400
300
200
100
0
f
Fold change of VEGF mRNA
21%
1%
NS
NS
3
2
1
0
g
(kDa)
siCont
sieIF4G
sieIF4E
O2 (%)
21 21 21 1 1 1
26-
VEGF
170-
eIF4G
26-
eIF4E
130-
HIF1α
55-
Tubulin
VEGF/Tubulin (%)
21%
1%
****
****
**
*
200
150
100
50
0

Supplementary Figure 5 The 4EHP and TRS interaction is critical for translation initiation of mRNAs required for vascular development. **a** Effect of TRS, 4EHP, eIF4G or eIF4E on cellular VEGF protein levels evaluated by immunoblotting in siTRS-, si4EHP-, sieIF4G-, or sieIF4E-transfected 293T cells (left) with band intensity normalized against tubulin (right). **b** Effect of suppressing TRS, 4EHP, eIF4G or eIF4E on *VEGF* transcription evaluated by RT-PCR analysis in 293T cells. **c** Effect of TRS, 4EHP, eIF4G or eIF4E on cellular ANG protein levels determined as in **a**. **d** Effect of TRS, 4EHP, eIF4G or eIF4E suppression on *ANG* transcripts analyzed as in **b**. **e** Protein levels of VEGF in 293T cells transfecting siRNAs against TRS or 4EHP cultured under normoxia (21% O_2) or hypoxia (1% O_2; left) with VEGF band intensity normalized against tubulin (right). **f** Induction of *VEGF* transcripts in response to normoxia (21% O_2) or hypoxia (1% O_2) measured by RT-PCR analysis in 293T cells. **g** VEGF protein levels in 293T cells transfected with siRNAs against eIF4G or eIF4E cultured under normoxia (21% O_2) or hypoxia (1% O_2; left) with VEGF band intensity normalized against tubulin (right). siCont, non-targeting control siRNA. *, $p < 0.05$; **, $p < 0.01$; ***, $p < 0.001$; ****$p < 0.0001$; NS, not significant vs. control group. Values are means ± SD of three independent experiments.

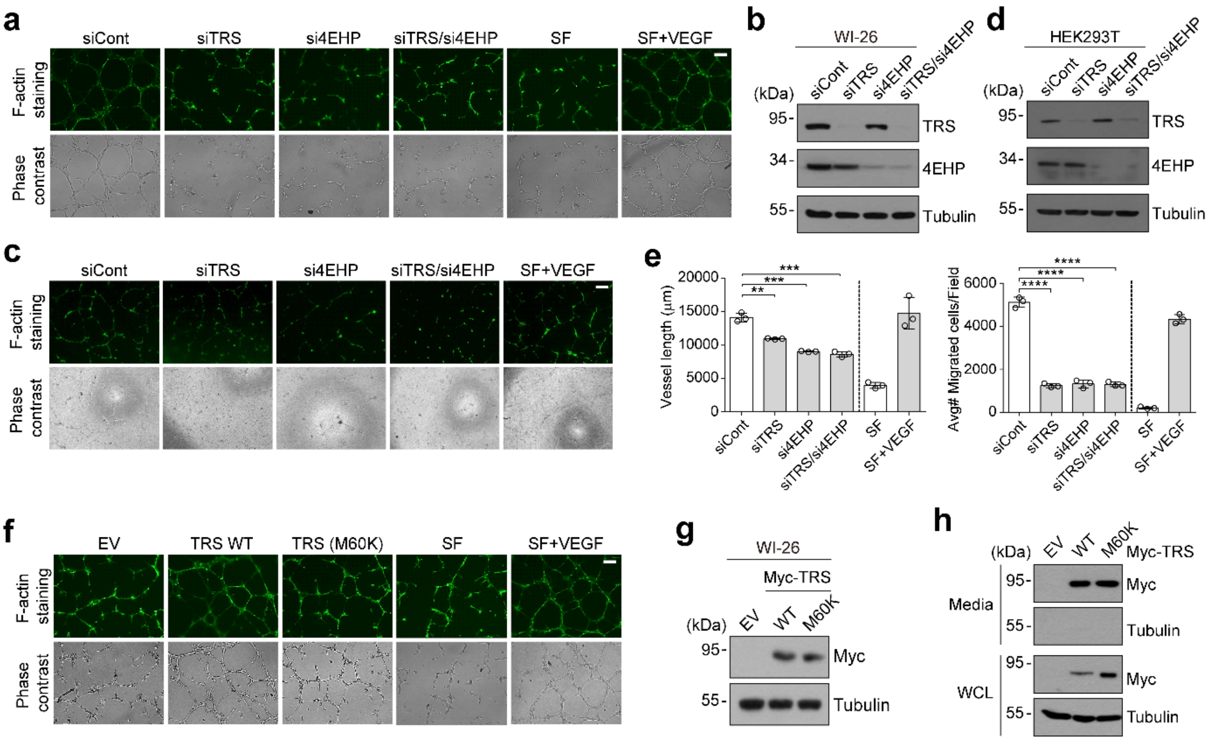

Supplementary Figure 6 Significance of intracellular TRS and 4EHP in vascular tube formation. **a**–**d** Effects of TRS and/or 4EHP suppression on endothelial cell tube formation. The effects of knock-down of TRS or 4EHP or TRS/4EHP with its specific siRNAs in WI-26 cells (**b**) and HEK293T cells (**d**) were determined by immunoblotting with each antibody. Culture medium from WI-26 (**a**) or 293T (**c**) cells transiently transfected with siRNAs against TRS, 4EHP, TRS/4EHP or a non-targeting control (siCont) was applied to HUVECs. Cells were subsequently plated on growth factor-reduced Matrigel to form capillary tubes, which were visualized by immunofluorescence microscopy of F-actin (upper), and by phase contrast microscopy (lower). Scale bar = 250 μm. **e** Quantification of total tube lengths observed by F-actin immunofluorescence microscopy in **c** using ImageJ (left). Trans-well migration assays were performed with 293T cell culture media-treated HUVECs. Migrated cells were counted from randomly chosen fields using phase contrast microscopy (right). **, *p* <0.01; ***, *p* <0.001; ****, *p* <0.0001 vs. control group. Values

are means ± SD of three independent experiments. **f** Effects of WT TRS or the 4EHP-binding-defective M60K mutant on endothelial cell tube formation. Culture medium from WI-26 cells transiently transfected with WT Myc-TRS or the M60K mutant was used to treat HUVECs, which were subsequently plated on growth factor-reduced Matrigel to form capillary tubes. Results are displayed as described in **a**, **c**. EV, empty vector. Scale bar = 250 μm. VEGF (10 ng mL^{-1}) served as a positive control. SF, serum-free medium (**a**, **c**, **e**, **f**). **g** Immunoblot analysis of Myc-TRS WT and Myc-TRS M60K mutant expressed in WI-26 cells using anti-Myc antibody. **h** Secretion levels of WT TRS and its M60K mutant. WI-26 cells were transfected with WT Myc-TRS or the M60K mutant for 24 h and subsequently incubated in SF media for 16 h. The amount of secreted and intracellular TRS was evaluated by immunoblotting. Data are representative of at least three experiments, each with similar results (**g**, **h**).

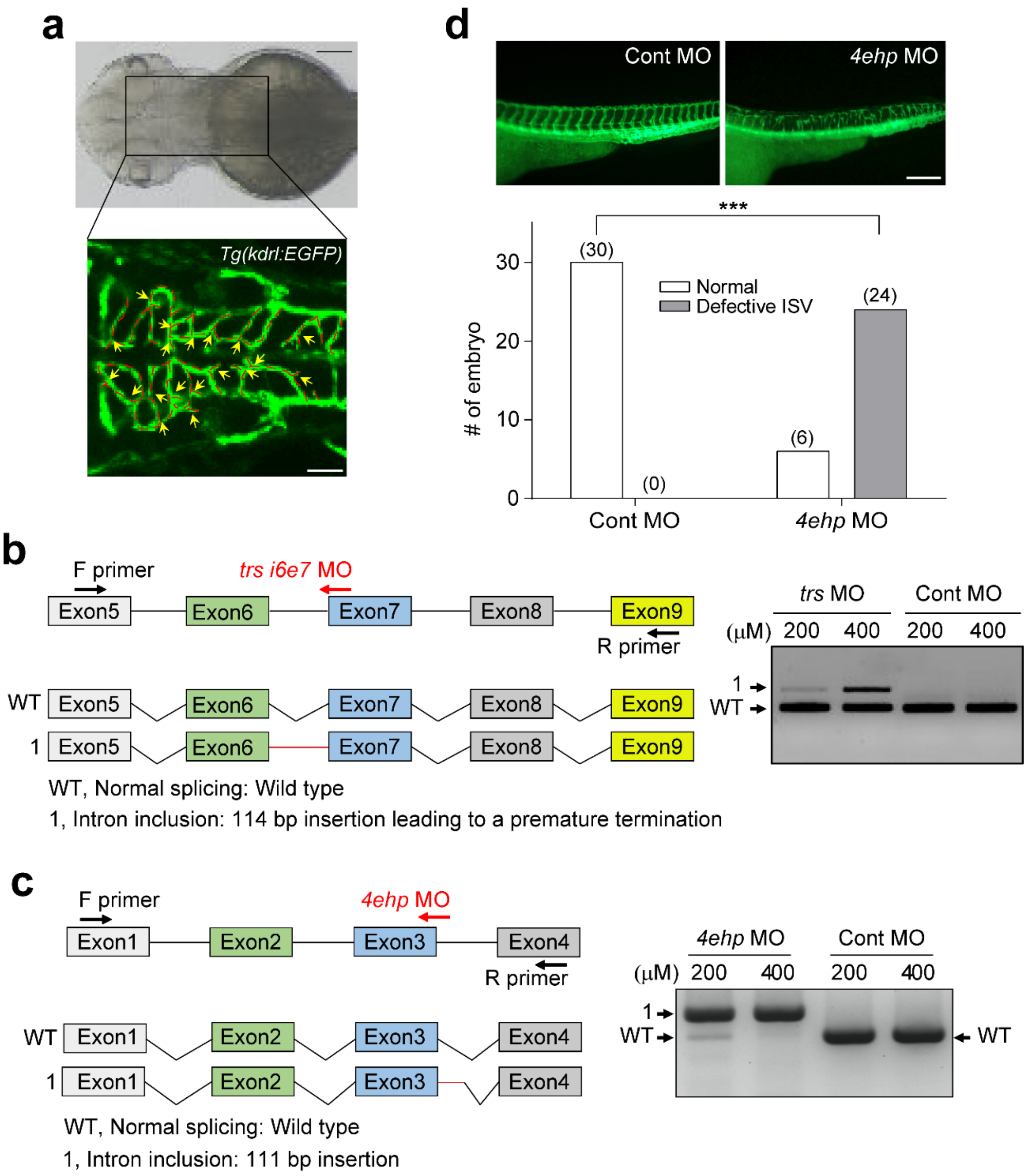

Supplementary Figure 7 Validation of TRS and 4EHP suppression in zebrafish. **a** Formation of stereotypical central arteries (CtAs) in the developing hindbrain of zebrafish embryos. (upper) Dorsal view bright-field image of the brain of zebrafish embryos captured at 52 h post-fertilization (hpf). The boxed region of the *Tg*(*kdrl:EGFP*) embryo is the area imaged using confocal microscopy. Scale bar = 100 μm. (lower) Representative z-projected fluorescence image of a *Tg*(*kdrl:EGFP*) embryo at 52 hpf. Using this image, the length (red dotted lines) and branching points

(yellow arrows) of CtA vessels were measured using ImageJ. Scale bar = 50 μm. **b** Validation of TRS knock-down by RT-PCR using *trs i6e7* MO. The location of the morpholino (MO) used to block the splicing of *trs* transcripts and primers used for RT-PCR are indicated in the genomic structure of zebrafish *trs*. Injection of different amounts of MO (200 and 400 μM) resulted in an additional single RT-PCR band due to aberrant splicing (band 1). Sequencing of the aberrant PCR band 1 revealed the 114 bp intron inclusion that leads to premature translational termination upon *trs i6e7* MO injection. Cont MO, control MO. **c** Validation of zebrafish *4ehp* knock-down by RT-PCR. The locations of the MO used to block *4ehp* splicing and primers used for RT-PCR are indicated in the genomic structure of zebrafish *4ehp*. Injection of different amounts of the MO shifted the RT-PCR band due to aberrant splicing (band 1). Sequencing of the aberrant PCR band 1 revealed the 111 bp intron inclusion. **d** Trunk vessel defects upon *4ehp* knock-down. (upper) Trunk vessels of control- (left) and *4ehp* MO-treated zebrafish (right) shown in lateral views. (lower) Angiogenic defects of intersegmental vessels (ISVs) in the developing trunk are more prevalent in *4ehp*-suppressed fish than in controls (Cont) at 52 hpf. Scale bar = 200 μm. Quantified data are shown in a bar graph. Numbers of analyzed zebrafish embryos are shown in bars. ***, p <0.001 vs. control group. Values are means ± SD.

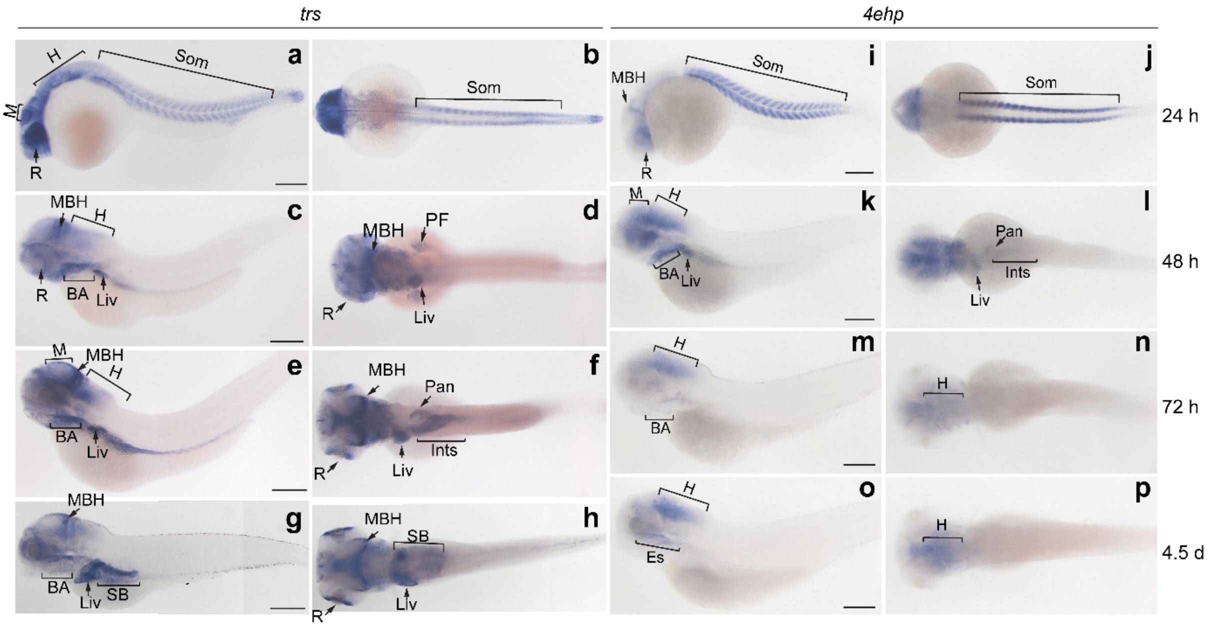

Supplementary Figure 8 Spatiotemporal expression of zebrafish *trs* and *4ehp* during embryogenesis by whole-mount RNA *in situ* hybridization. **a**–**h** *trs* expression. **i**–**p** *4ehp* expression at 24 hpf (**a**, **b**, **i**, **j**), 48 hpf (**c**, **d**, **k**, **l**), 72 hpf (**e**, **f**, **m**, **n**) and 4.5 dpf (**g**, **h**, **o**, **p**). Lateral view (**a**, **c**, **e**, **g**, **i**, **k**, **m**, **o**). Dorsal view (**b**, **d**, **f**, **h**, **j**, **l**, **n**, **p**). All images are oriented with the rostral to the left. Both *trs* and *4ehp* are expressed in the hindbrain and somites relevant to vascular defects upon morpholino knock-down during embryogenesis, in addition to several other developing tissues, indicating diverse roles. BA, branchial arches; H, hindbrain; Ints, intestine; Liv, liver; MBH, midbrain-hindbrain boundary; M, midbrain; Pan, pancreas; PF, pectoral fin; R, retina; Es: esophagus. Scale bars = 200 μm.

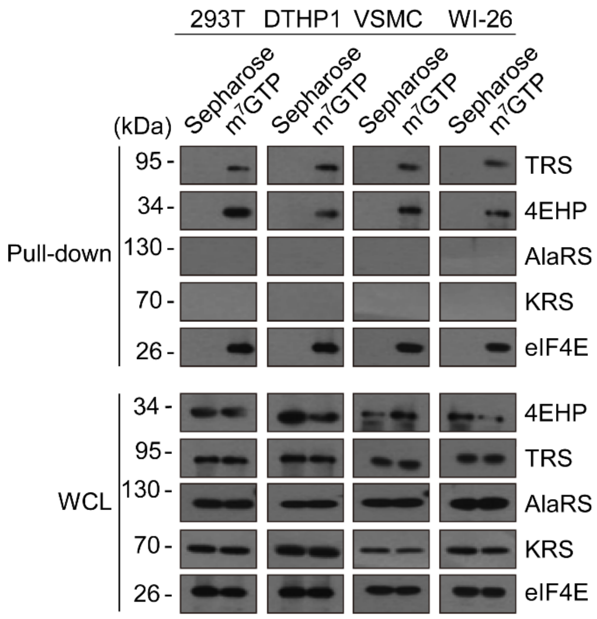

Supplementary Figure 9 Cap structure-binding assay of endogenous 4EHP and TRS in lysates of various cell lines. Equal amounts of total extracted proteins were incubated with Protein A-Sepharose or cap analog m^7GTP-Sepharose, followed by immunoblotting with anti-TRS, 4EHP, AlaRS, KRS, and eIF4E antibodies. DTHP1, differentiated THP1; WCL, whole cell lysate. Data are representative of at least three experiments, each with similar results.

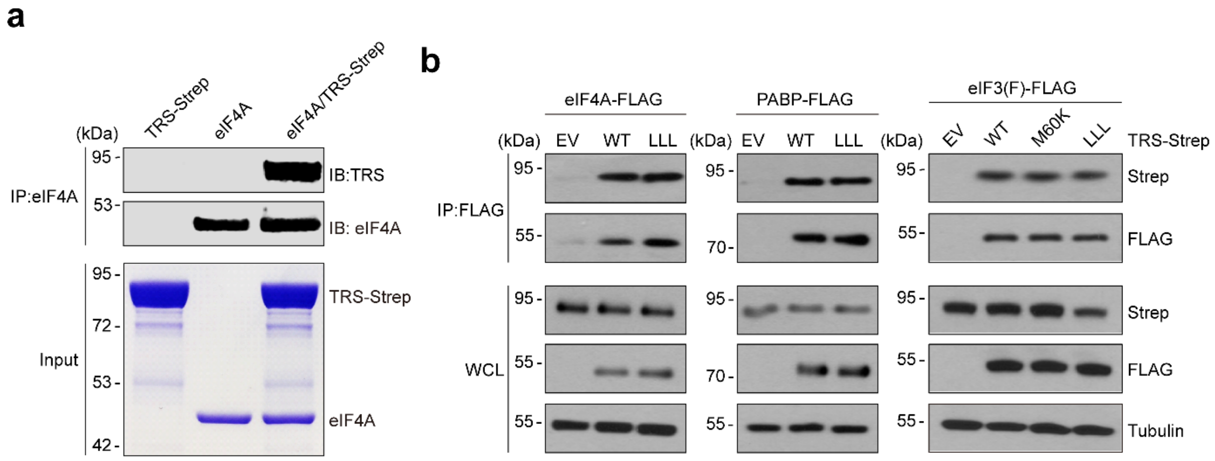

Supplementary Figure 10 Interaction between TRS and translation initiation factors. **a** Immunoassay showing direct interaction between TRS and eIF4A. Purified TRS-Strep and eIF4A proteins were incubated and immunoprecipitated with anti-eIF4A antibody, followed by further incubation with Protein A/G PLUS-agarose beads. Immunoprecipitates were intensively washed and co-precipitated TRS with eIF4A was analyzed by immunoblotting with anti-TRS antibody. Input proteins were separated by SDS-PAGE and stained with Coomassie Brilliant Blue. **b** Immunoassay showing that the interaction of eIF4A, PABP or eIF3 subunit F with TRS is not affected by the mutation (LLL mutant) of critical residues in the TRS ABD site for binding to the anticodon^{Thr}-like loop. eIF4A-FLAG, PABP-FLAG, or eIF3 subunit F-FLAG was co-expressed with WT, LLL, or M60K mutant TRS-Strep in 293T cells, immunoprecipitated with anti-FLAG antibody, and co-precipitated TRS proteins with each FLAG-tagged protein were determined by immunoblotting with anti-Strep antibody. Note that the M60K mutant TRS is defective for interaction with 4EHP. EV, empty vector; LLL, R663L/E680L/R689L mutant TRS; WCL, whole cell lysate. Data are representative of at least three experiments, each with similar results.

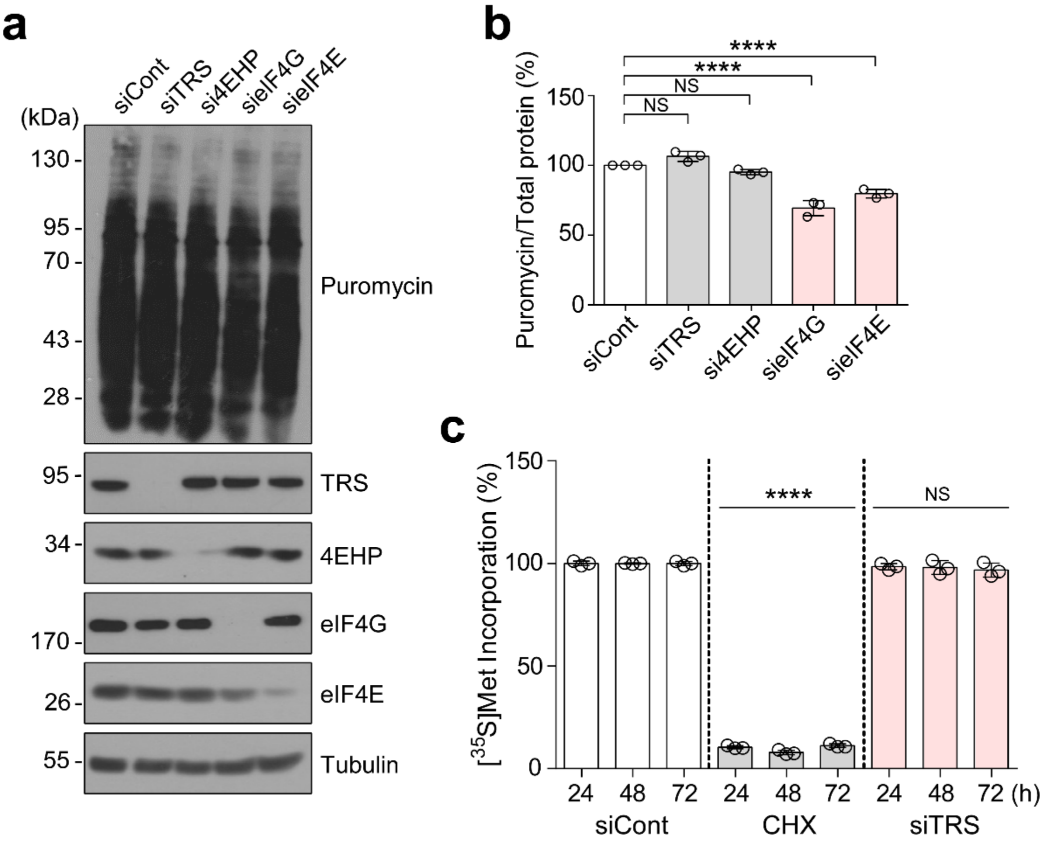

Supplementary Figure 11 Effects of suppression of eukaryotic translation initiation factors on *de novo* protein synthesis. **a** TRS, 4EHP, eIF4G or eIF4E was suppressed with their specific siRNAs for 48 h in 293T cells followed by treatment with puromycin (1 μM) for 30 min. *De novo* global protein synthesis was then monitored by immunoblotting cell lysates with anti-puromycin antibody. **b** Bar graph of the ratio of *de novo* protein synthesis vs. global protein synthesis determined as a percentage. **c** Protein synthesis was measured in TRS knocked- down 293T cells at the indicated times by [^{35}S]-Met incorporation assay. Cycloheximide (CHX) treatment for 30 min was used as a positive control. siCont, non-targeting control siRNA. ****, p <0.0001; NS, not significant vs. control group. Values are means ± SD of three independent experiments.

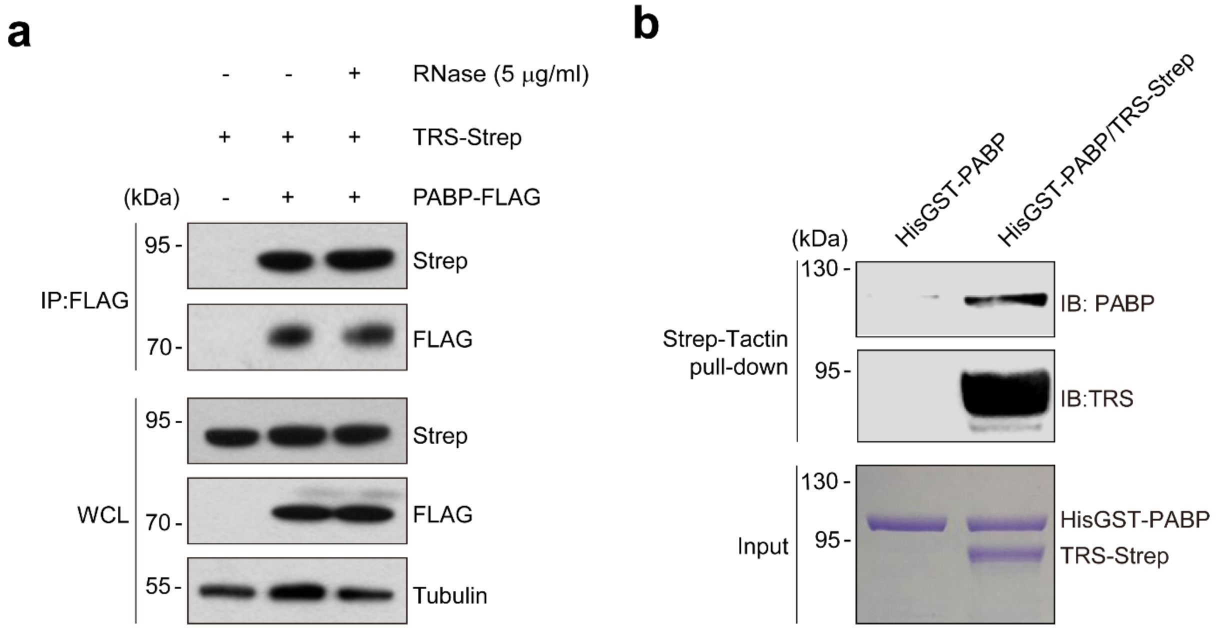

Supplementary Figure 12 Interaction between TRS and PABP. **a** Immunoassay showing that the interaction between TRS and PABP is not mediated by mRNA. PABP-FLAG was co-expressed with TRS-Strep in 293T cells. PABP-FLAG was immunoprecipitated with anti-FLAG antibody and incubated with RNase (10 μg mL^{-1}) at 37°C for 1 h before washing, and co-precipitated TRS with PABP was determined by immunoblotting with anti-Strep antibody. **b** *In vitro* pull-down assay of TRS-Strep and His-GST-PABP. Purified TRS-Strep and His-GST-PABP were incubated and pulled down with Strep-Tactin beads, and co-precipitated PABP was eluted from the resin and detected by immunoblotting with anti-PABP antibody. Input proteins were visualized by Coomassie staining. Data are representative of at least three experiments, each with similar results.

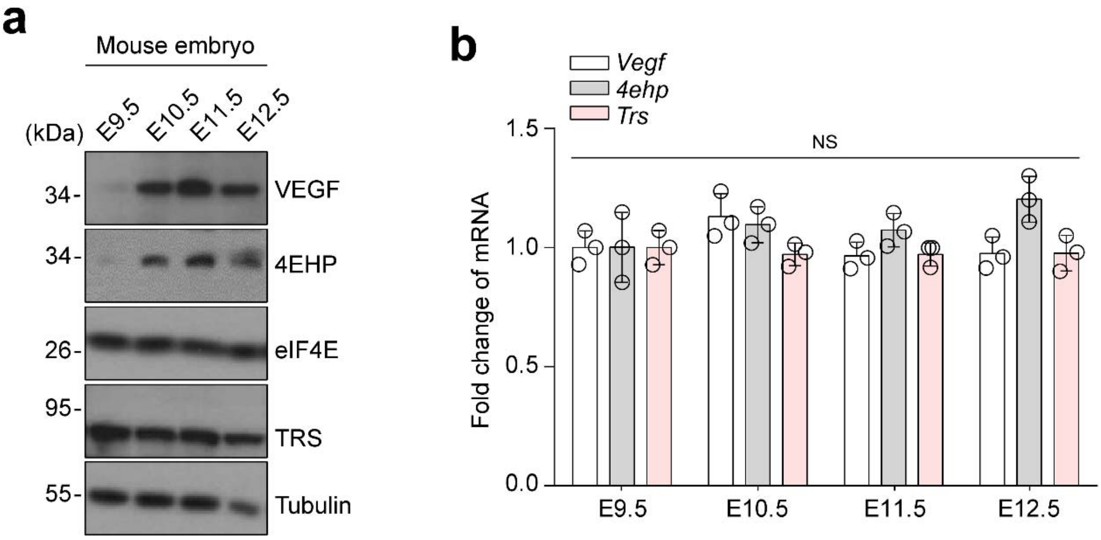

Supplementary Figure 13 Changes in VEGF, 4EHP, and TRS protein and mRNA levels at different developmental stages. **a**, **b** Cellular protein levels of VEGF, 4EHP, eIF4E, and TRS (**a**) and mRNA levels of *Vegf*, *4ehp*, and *Trs* (**b**) determined by immunoblotting with specific antibodies and RT-PCR, respectively, in whole mouse embryos at different developmental stages. NS, not significant vs. control group. Values are means ± SD of three independent experiments.

Fig. 1b

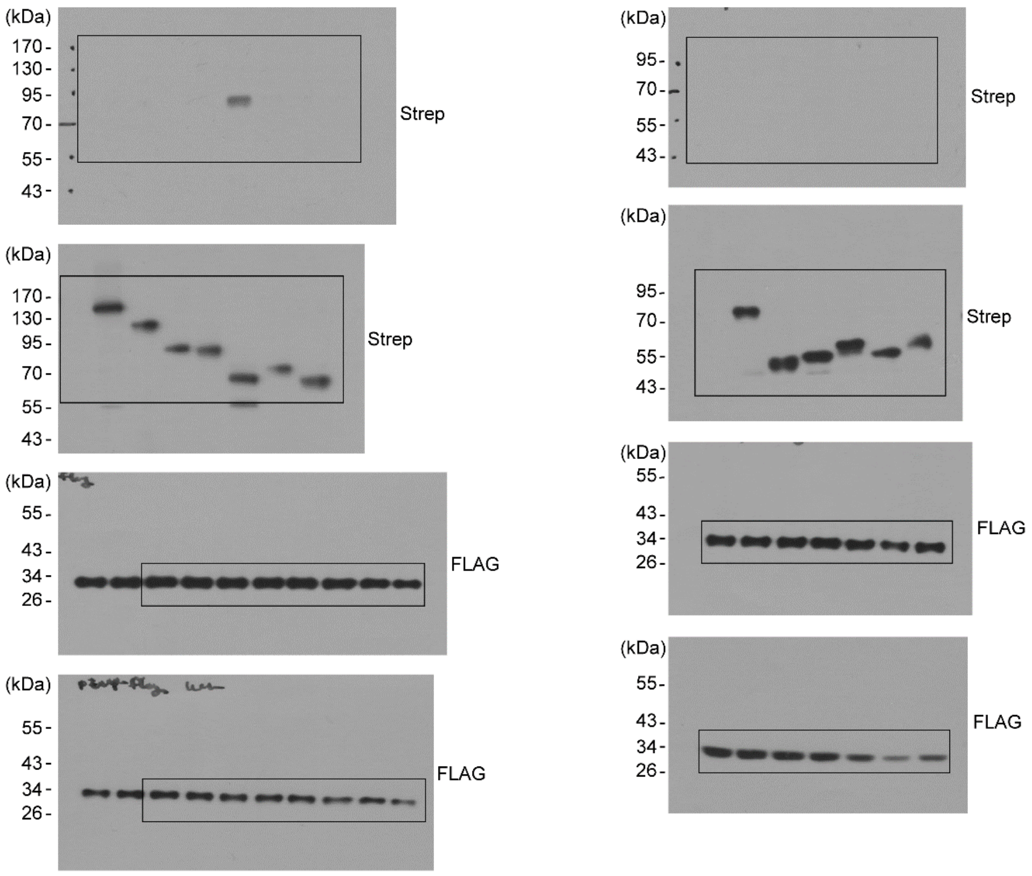

Fig. 1g

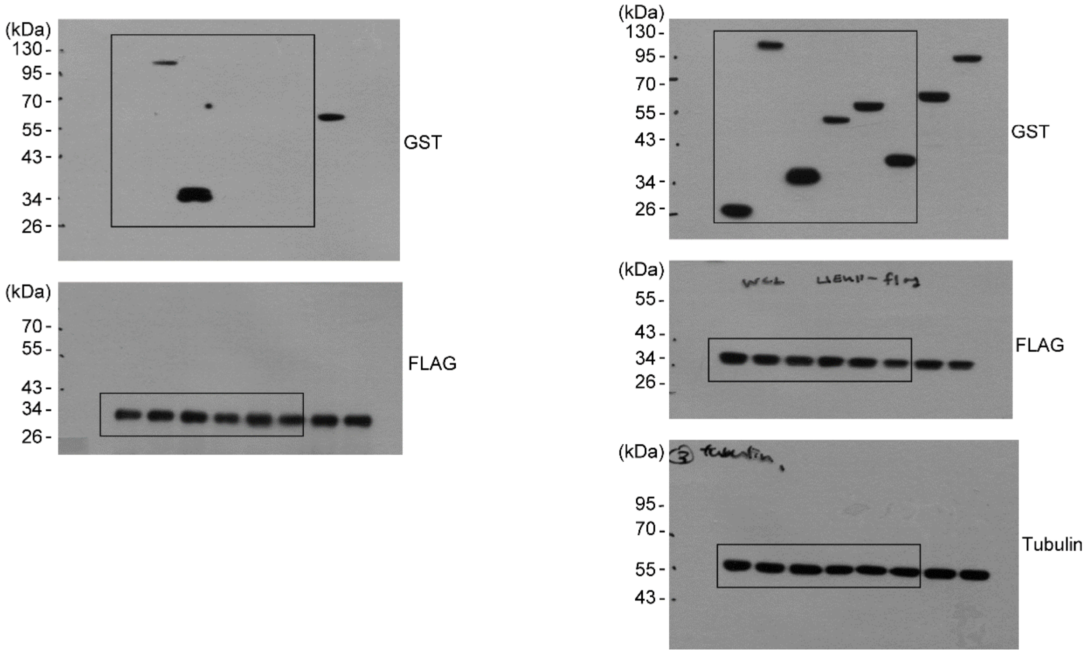

Supplementary Figure 14 Uncropped and unprocessed immunoblots. Line boxes indicate the cropped areas shown in the corresponding figures.

Fig. 6a

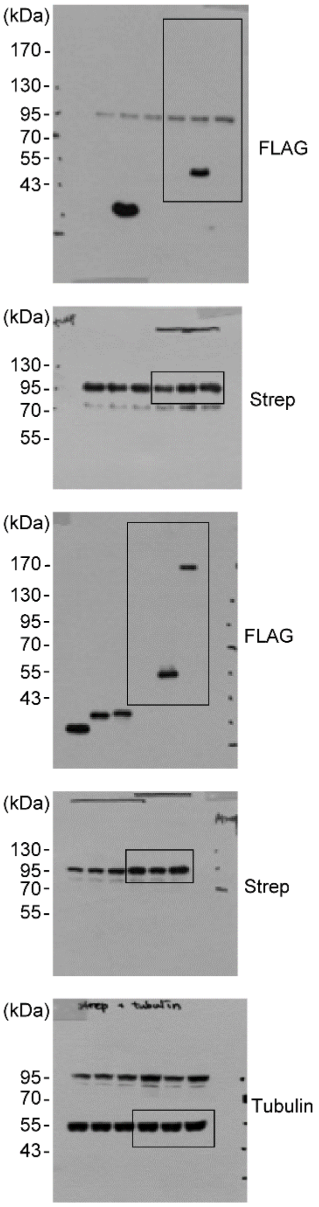

Fig. 6b

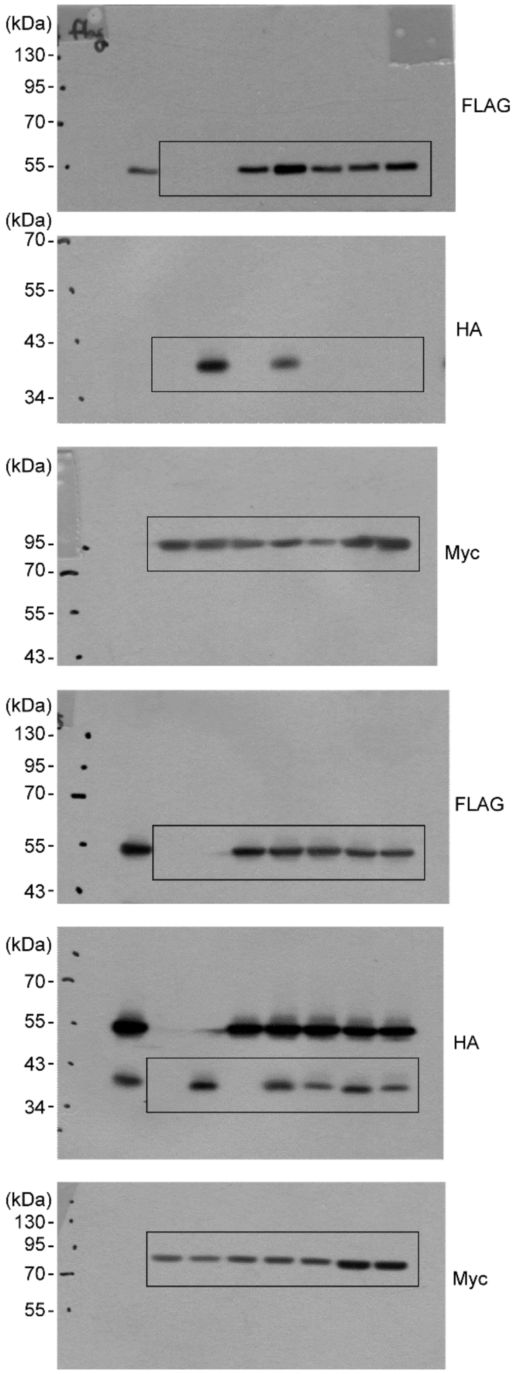

Supplementary Figure 15 Uncropped and unprocessed immunoblots. Line boxes indicate the cropped areas shown in the corresponding figures.

Fig. 6c

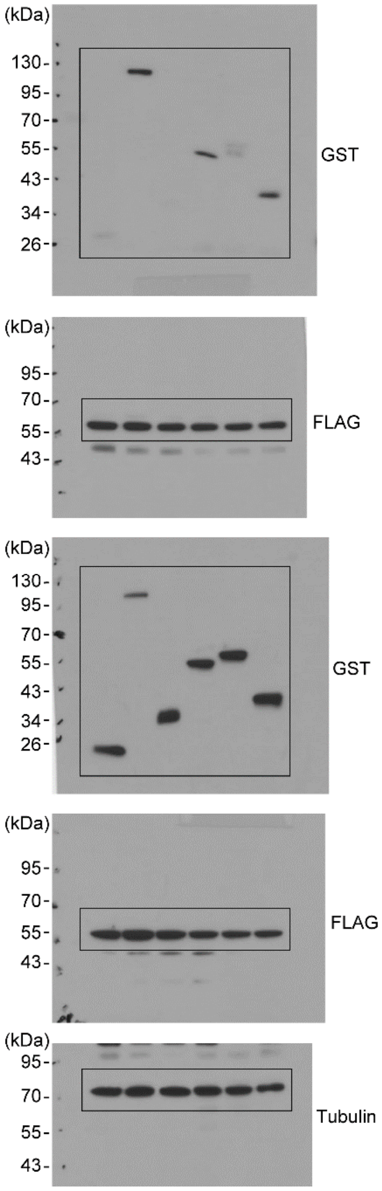

Fig. 7a

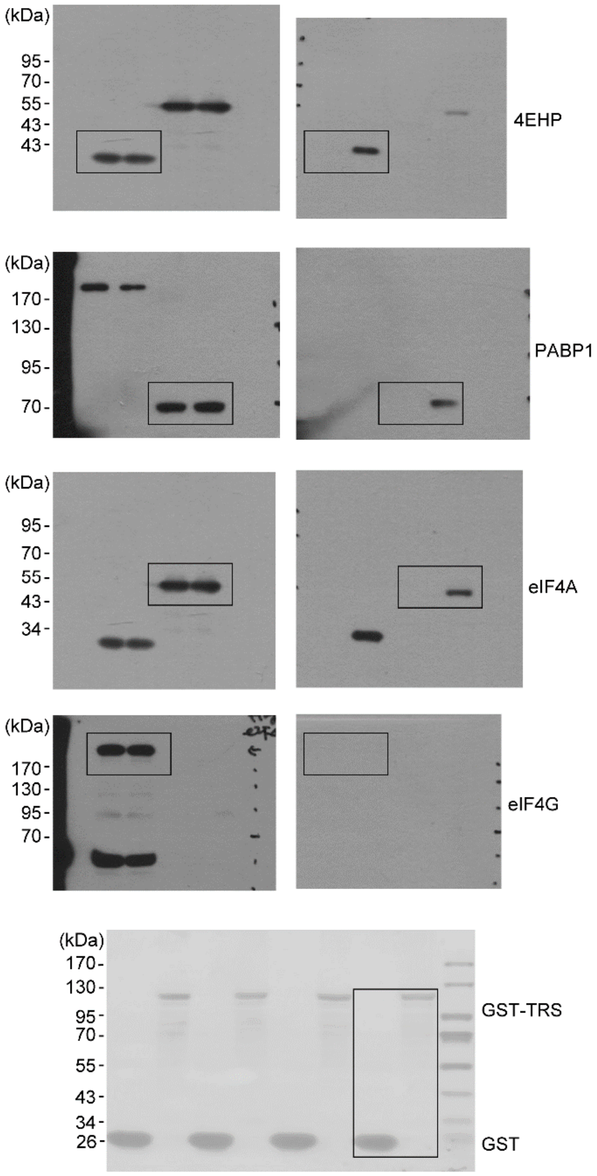

Supplementary Figure 16 Uncropped and unprocessed immunoblots. Line boxes indicate the cropped areas shown in the corresponding figures.

Fig. 7b

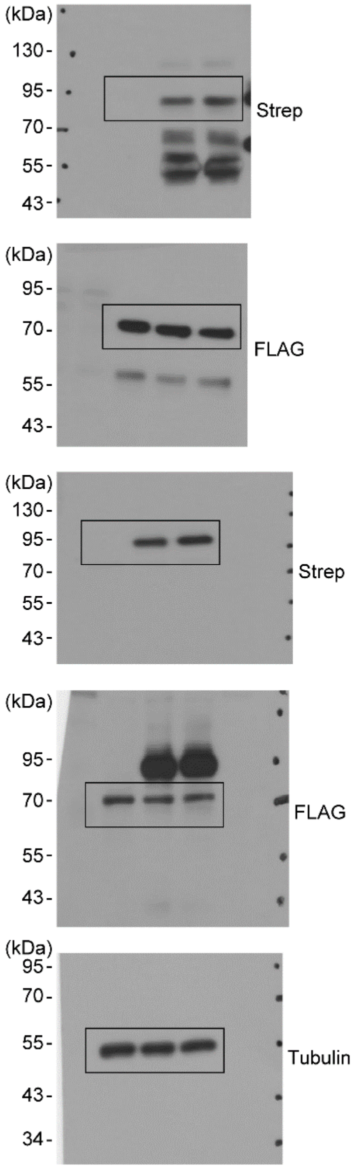

Fig. 7c

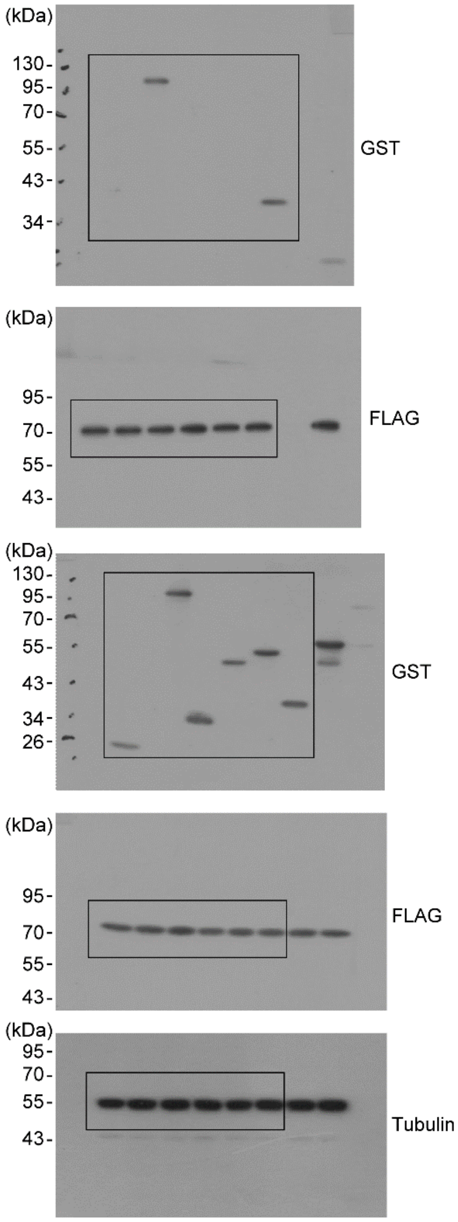

Supplementary Figure 17 Uncropped and unprocessed immunoblots. Line boxes indicate the cropped areas shown in the corresponding figures.

Supplementary Table 1 Data collection and refinement statistics

	TRS (UNE-T)-4EHP complex
**Data collection**	
Space group	$P2_12_12_1$
Cell dimensions	
a, b, c (Å)	49.37, 60.57, 85.24
α, β, γ (°)	$\alpha = \beta = \gamma = 90$
Wavelength	0.97934
Resolution (Å)	50–1.90 (1.97–1.90)*
No. total reflections	146,691
No. unique reflections	20,761 (2,014)
Redundancy	7.1 (7.1)
Completeness (%)	99.9 (100)
R_{sym} (%)	9.4 (55.3)
$I/\sigma I$	21.1 (13.4)
**Refinement**	
Resolution (Å)	25.8–1.90 (1.95–1.90)
No. reflections	19,654
No. reflections used for R_{free}	1,062
R_{work}/R_{free}	0.16/0.20
R.m.s. deviation	
Bond lengths (Å)	0.007
Bond angles (°)	0.990
No. atoms[a]	
Protein	1,577
Waters	177
Average *B*-factors (Å^2)	
4EHP (Chain A) / TRS (Chain B)	27.40/39.05
Waters	42.05
Ramachandran statistics (%)	
Favored regions	98.38
Additionally allowed regions	1.62
PDB accession code	5XLN

*Values in parentheses are for the highest resolution shell.
[a]Total number of atoms per asymmetric unit.

Supplementary Table 2 Materials used in this study

REAGENT or RESOURCE	SOURCE	IDENTIFIER
**Antibodies**		
Mouse monoclonal anti-Strep	IBA	Cat#2-1509-001
Mouse monoclonal anti-FLAG M2	Sigma-Aldrich	Cat#F3165; RRID: AB_259529
Mouse monoclonal anti-alpha-Tubulin	Sigma-Aldrich	Cat#T6074; RRID: AB_477582
Mouse monoclonal anti-ThrRS (C-3)	Santa Cruz Biotechnology	Cat#sc-166146; RRID: AB_2200663
Rabbit monoclonal anti-ThrRS (H-100)	Santa Cruz Biotechnology	Cat#sc-98543; RRID: AB_1569132
Mouse monoclonal anti-ThrRS (TARSF8H3)	Santa Cruz Biotechnology	Cat#sc-81409; RRID: AB_1129970
Rabbit polyclonal anti-EIF4E2 (N1C3)	GeneTex	Cat#GTX103977; RRID: AB_2036842
Mouse monoclonal anti-HA-probe (F-7)	Santa Cruz Biotechnology	Cat#sc-7392; RRID: AB_627809
Mouse monoclonal anti-GST (26H1)	Cell Signaling Technology	Cat#2624; RRID: AB_10692101
Mouse monoclonal anti-c-Myc (9E10)	Santa Cruz Biotechnology	Cat#sc-40; RRID: AB_390912
Mouse monoclonal anti-V5	Thermo Fisher	Cat#R961-25; RRID: AB_2556565
Rabbit monoclonal anti-eIF4GI (D6A6)	Cell Signaling Technology	Cat#8701; RRID: AB_11178378
Mouse monoclonal anti-eIF-4E	BD Biosciences	Cat#610269; RRID: AB_397664
Rabbit monoclonal anti-eIF4A (C32B4)	Cell Signaling Technology	Cat#2013; RRID: AB_2097363
Mouse monoclonal anti-Puromycin (12D10)	Millipore	Cat#MABE343
Rabbit polyclonal anti-AlaRS (H-268)	Santa Cruz Biotechnology	Cat#sc-98547; RRID: AB_1562673
Mouse monoclonal anti-AlaRS (M6-P2E5)	Santa Cruz Biotechnology	Cat#sc-81712; RRID: AB_1127445
Mouse IgG_1	Santa Cruz Biotechnology	Cat#sc-3877; RRID: AB_737222
Mouse monoclonal anti-ProRS (A-2)	Santa Cruz Biotechnology	Cat#sc-393505; RRID: AB_? – not found
Mouse monoclonal anti-IleRS (D-9)	Santa Cruz Biotechnology	Cat#sc-271826; RRID: AB_10709166
Rabbit polyclonal anti-VEGF (A-20)	Santa Cruz Biotechnology	Cat#sc-152; RRID: AB_2212984
Mouse monoclonal anti-ANG I (C-1)	Santa Cruz Biotechnology	Cat#sc-74528; RRID: AB_2227157
Rabbit polyclonal anti-HIF1-alpha	Bethyl	Cat#A300-286A; RRID: AB_2117114
Rabbit polyclonal anti-LysRS	Abcam	Cat#ab31532; RRID: AB_776117
Rabbit polyclonal anti-PABP	Abcam	Cat#ab21060; RRID: AB_777008
Rabbit polyclonal anti-eIF3A	Novus	Cat#NBP1-18891; RRID: AB_1625664
Rabbit polyclonal anti-eIF3B	Bethyl	Cat#A301-761A; RRID: AB_1210995
Rabbit polyclonal anti-eIF3C	Bethyl	Cat#A300-377A; RRID: AB_2096755

Rabbit polyclonal anti-eIF3D	Bethyl	Cat#A301-758A; RRID: AB_1210970
Rabbit polyclonal anti-eIF3E	Bethyl	Cat#A302-985A; RRID: AB_10749034
Rabbit polyclonal anti-eIF3F	Bethyl	Cat#A303-005A; RRID: AB_10748371
Rabbit polyclonal anti-eIF3G	Bethyl	Cat#A301-757A; RRID: AB_1210991
Rabbit polyclonal anti-eIF3H	Bethyl	Cat#A301-754A; RRID: AB_1210983
Rabbit polyclonal anti-eIF3I	Biolegend	Cat#646701; RRID: AB_1732047
Rabbit polyclonal anti-eIF3J	Biolegend	Cat#638401; RRID: AB_2293350
Rabbit polyclonal anti-eIF3K	Novus	Cat#NB100-93304; RRID: AB_1236913
Rabbit polyclonal anti-eIF3L	GeneTex	Cat#GTX120119; RRID: AB_11169154
Rabbit polyclonal anti-eIF3M	Novus	Cat#NBP1-56654; RRID: AB_11030684
Alexa Fluor 488 Phalloidin	Thermo Fisher	Cat#A12379
Goat anti-mouse-HRP conjugated	Thermo Fisher	Cat#31430
Goat anti-rabbit-HRP conjugated	Thermo Fisher	Cat#31460
Alexa Flour 594	Thermo Fisher	Cat#A21201
Alexa Flour 647	Thermo Fisher	Cat#A27040
**Bacterial strains**		
E. coli: BL21 (DE3)	Novagen	Cat#69450
E. coli: BL21-CodonPlus (DE3)-RIPL	Agilent	Cat#230280
E. coli: BL21-star (DE3)	Invitrogen	Cat#6010-03
E. coli: Rosetta-gami (DE3) pLysS	Novagen	Cat#71057
E. coli: BLR (DE3)	Novagen	Cat#69053
E. coli: C43 (DE3)	Lucigen	Cat#60446
**Chemicals and recombinant proteins**		
DAPI	Thermo Fisher	Cat#62248
FuGENE HD	Roche	Cat#E2311
Glutaraldehyde	Sigma	Cat#G5882
Glycogen	Invitrogen	Cat#10814010
Gel Mount	Biomeda	Cat#M01
Hematoxylin solution	Sigma Aldrich	Cat#MHS16
Lipofectamine 2000	Thermo Fisher	Cat#11668030
X-tremeGENE™ HP DNA Transfection Reagent	Roche	Cat# XTGHP-RO
PEG8000	Sigma-Aldrich	Cat#P2139
Phorbol 12-myristate 13-acetate	Sigma-Aldrich	Cat#P1585
Puromycin	Thermo Fisher	Cat#A1113802
Recombinant Human VEGF	R&D Systems	Cat#293-VE-010
rTEV protease, recombinant	Invitrogen	Cat#10127-017
TurboFect	Thermo Fisher	Cat#R0531
1-phenyl 2-thiourea, PTU	Sigma-Aldrich	Cat#P-7629
d-Desthiobiotin	Sigma-Aldrich	Cat#D1411
Protease inhibitor	Calbiochem	Cat#535140
Phosphatase inhibitor	Thermo	Cat#78427
Cycloheximide	Sigma-Aldrich	Cat#01810
Trichloroacetic acid	Sigma-Aldrich	Cat#T0699

**Commercial assay kits**		
QuikChange II Site-Directed Mutagenesis Kit	Agilent	Cat#200523
Dynabeads Co-Immunoprecipitation Kit	Thermo Fisher	Cat#14321D
TruSeq RNA Sample Preparation Kit v2	Illumina	Cat#RS-122-2101
Luciferase Assay System	Promega	Cat#E1500
Renilla Luciferase Assay System	Promega	Cat#E2810
Human VEGF Quantikine ELISA kit	R&D Systems	Cat#DVE00
Human Angiogenin Quantikine ELISA kit	R&D Systems	Cat#DAN00
mMESSAGE mMACHINE SP6 Transcription Kit	Ambion	Cat#AM1340
**Cell lines**		
Human: HEK293T/17 cell line	ATCC	Cat#CRL-11268; RRID: CVCL_1926
Human: WI-26 VA4 cells	ATCC	Cat#CCL-95.1
Hamster: CHO-K1 cells	KCLB	Cat#10061
Drosophila melanogaster: S2 cell line	DGRC, the Indiana University	N/A
Human: THP-1 cells	KCLB	Cat#40202
Human: VSMCs	ScienCell	Cat#1100
Human: HUVECs	ATCC	Cat#PCS-100-010
**Organisms**		
Zebrafish: *Tg(kdrl:EGFP)*s843; s843Tg	Jin and Stainier, 2005	ZFIN: ZDB-ALT-050916-14
Mouse: C57BL/6J	The Jackson Laboratory	Stock No.: 000664; RRID:IMSR_JAX:000664
**Oligonucleotides**		
Non-targeting Control siRNA	Dharmacon	Cat#D-001810
siRNA targeting TRS (SMARTpool)	Dharmacon	Cat#L-011789
siRNA targeting AlaRS (SMARTpool)	Dharmacon	Cat#L-011565
siRNA targeting EIF4E2 (SMARTpool)	Dharmacon	Cat#L-019870
siRNA targeting EIF4E (SMARTpool)	Dharmacon	Cat#L-003884
Stealth RNAi siRNA Negative Control, Med GC	Thermo Fisher	Cat#12935300
siRNA targeting EIF4G (Stealth RNAi)	Thermo Fisher	Cat#10620318
TRS Y53F Forward GTTGAATCCTTGGCCTGAATTTATTTACACACGTCTTGAGA	This paper	N/A
TRS Y53F Reverse TCTCAAGACGTGTGTAAATAAATTCAGGCCAAGGATTCAAC	This paper	N/A
TRS Y55F Forward GTTGAATCCTTGGCCTGAATATATTTTCACACGTCTTGAGA	This paper	N/A
TRS Y55F Reverse TCTCAAGACGTGTGAAAATATATTCAGGCCAAGGATTCAAC	This paper	N/A
TRS M60K Forward CATGTTCTGCTTTTAGTATATTATACTTCTCAAGACGTGTGTAAATATATTC	This paper	N/A
TRS M60K Reverse GAATATATTTACACACGTCTTGAGAAGTATAATATACTAAAAGCAGAACATG	This paper	N/A

TRS I63V Forward TATTTACACACGTCTTGAGATGTATAATGTACTAAAAGCAGAACATGATTC	This paper	N/A
TRS I63V Reverse GAATCATGTTCTGCTTTTAGTACATTATACATCTCAAGACGTGTGTAAATA	This paper	N/A
TRS C413S Forward GGCAGTGTCCTGGGCTGTTCATGGGTTTCAG	This paper	N/A
TRS C413S Reverse CTGAAACCCATGAACAGCCCAGGACACTGCC	This paper	N/A
TRS R663L Forward CTGTACATTGAATAAAAAGATTCGAAATGCACAGTTAGCACAGTA	This paper	N/A
TRS R663L Reverse TACTGTGCTAACTGTGCATTTCGAATCTTTTTATTCAATGTACAG	This paper	N/A
TRS E680L Forward CATTTTAGTTGTTGGTGAAAAACTGAAAATCAGTGGCACTGTTAAT	This paper	N/A
TRS E680L Reverse ATTAACAGTGCCACTGATTTTCAGTTTTTCACCAACAACTAAAATG	This paper	N/A
TRS R689L Forward ATCAGTGGCACTGTTAATATCCTGACAAGAGACAATAAGGTCCAC	This paper	N/A
TRS R689L Reverse GTGGACCTTATTGTCTCTTGTCAGGATATTAACAGTGCCACTGAT	This paper	N/A
Zebrafish TRS D50I Forward GTTATAAATGGACAGGCGCTCAATAATGTACTGTGGTGGAGGACTC	This paper	N/A
Zebrafish TRS D50I Reverse GAGTCCTCCACCACAGTACATTATTGAGCGCCTGTCCATTTATAAC	This paper	N/A
Zebrafish TRS I55D Forward TACATTGATGAGCGCCTGTCCGATTATAACAAACTGAAGGAAGAG	This paper	N/A
Zebrafish TRS I55D Reverse CTCTTCCTTCAGTTTGTTATAATCGGACAGGCGCTCATCAATGTA	This paper	N/A
4EHP H54N Forward GTACTGCAGGGGATTCTCTGCCGGTCCAG	This paper	N/A
4EHP H54N Reverse CTGGACCGGCAGAGAATCCCCTGCAGTAC	This paper	N/A
4EHP F94W Forward TATAAAACCTCCACCACTGCTCCACAGAGGCAAAGGTGCC	This paper	N/A
4EHP F94W Reverse GGCACCTTTGCCTCTGTGGAGCAGTGGTGGAGGTTTTATA	This paper	N/A
4EHP L156K Forward AACCATGAACTGTTCCCCCTTCATGGCCAAAATGAGATTC	This paper	N/A

4EHP L156K Reverse<br>GAATCTCATTTTGGCCATGAAGGGGGAACAGTTCATGGTT	This paper	N/A
eIF3C Forward<br>CTAGCTAGCATGTCGCGGTTTTTCACCACC	This paper	N/A
eIF3C Reverse<br>GCTCTAGAGTAGGCCGTCTGAGACTGCTGC	This paper	N/A
eIF3D Forward<br>CTAGCTAGCATGGCAAAGTTCATGACACCCG	This paper	N/A
eIF3D Reverse<br>GCTCTAGAAGTTTCTTCCTCTTCTTCTTCCTCTTCTTC	This paper	N/A
eIF3E Forward<br>CTAGCTAGCATGGCGGAGTACGACTTGACTACTCG	This paper	N/A
eIF3E Reverse<br>GCTCTAGAGTAGAAGCCAGAATCTTGAGTTGCCC	This paper	N/A
eIF3F Forward<br>CTAGCTAGCATGGCCACACCGGCGGTACC	This paper	N/A
eIF3F Reverse<br>GCTCTAGACAGGTTTACAAGTTTTTCATTGAGTGCAATCT	This paper	N/A
eIF3G Forward<br>CTAGCTAGCATGCCTACTGGAGACTTTGATTCGA	This paper	N/A
eIF3G Reverse<br>GCTCTAGAGTTGGTGGACGGCTTGGCC	This paper	N/A
eIF3H Forward<br>CTAGCTAGCATGGCGTCCCGCAAGGAAGG	This paper	N/A
eIF3H Reverse<br>GCTCTAGAGTTGTTGTATTCTTGAAGAGCCTGGG	This paper	N/A
eIF3I Forward<br>CTAGCTAGCATGAAGCCGATCCTACTGCAG	This paper	N/A
eIF3I Reverse<br>GCTCTAGAAGCCTCAAACTCAAATTCGAAGTACT	This paper	N/A
eIF3J Forward<br>CTAGCTAGCATGGCGGCGGCGGCG	This paper	N/A
eIF3J Reverse<br>GCTCTAGACATGAAGTCTTCATAGTCTTGTACATATCCTCCATC	This paper	N/A
eIF3K Forward<br>CTAGCTAGCATGGCGATGTTTGAGCAGATGAGAG	This paper	N/A
eIF3K Reverse<br>GCTCTAGACTGGGAGGAGGCCATGATGCT	This paper	N/A
eIF3L Forward<br>CTAGCTAGCATGTCTTATCCCGCTGATGATTATG	This paper	N/A

eIF3L Reverse GCTCTAGAAGGTCTCTGTCCCATCTTCTTCAG	This paper	N/A
eIF3M Forward CTAGCTAGCATGAGCGTCCCGGCCTTC	This paper	N/A
eIF3M Reverse GCTCTAGAGGTATCAGAAAGACTCAAAAGGCTG	This paper	N/A
VEGFA_5’ UTR -1038 Forward TCGCGGAGGCTTGGGGCAGC	This paper	N/A
VEGFA_5’ UTR -685 Reverse CCTCGACTTCTCTCTGGAGCTC	This paper	N/A
VEGFA_5’ UTR -706 Forward GAGCTCCAGAGAGAAGTCGAGG	This paper	N/A
VEGFA_5’ UTR -352 Reverse GCGAGAACAGCCCAGAAGTTGG	This paper	N/A
VEGFA_5’ UTR -373 Forward CCAACTTCTGGGCTGTTCTCGC	This paper	N/A
VEGFA_5’ UTR +3 Reverse CATGGTTTCGGAGGCCCGACC	This paper	N/A
VEGFA_5’ UTR -18 Forward GGTCGGGCCTCCGAAACCATG	This paper	N/A
VEGFA_5’ UTR +306 Reverse GATGTTGGACTCCTCAGTGGGC	This paper	N/A
VEGFA_5’ UTR -749 Forward GGGGTACCTCGCGGAGGCTTGGGGCAGCCG	This paper	N/A
VEGFA_5’ UTR -749 Reverse GAAGATCTCGCGACTGGTCAGCTGCGGGATC	This paper	N/A
VEGFA_5’ UTR -167 Forward GGGGTACCCTGACGGACAGACAGACAGACAC	This paper	N/A
VEGFA_5’ UTR -167 Reverse GAAGATCTAGCGCGCGCGGCTGGAGCACTGT	This paper	N/A
VEGFA_5’ UTR -167 18bp_up Forward GGGGTACCGACACCGCCCCCAGCCCCAGCTAC	This paper	N/A
VEGFA_5’ UTR -167 18bp_down Reverse GAAGATCTACTGTCTGCGCACACCGCCGCCT	This paper	N/A
VEGFA_5’ UTR CGU_ Forward CTGTCTGCGCACGCCGCCGCCTCAC	This paper	N/A
VEGFA_5’ UTR CGU_ Reverse GTGAGGCGGCGGCGTGCGCAGACAG	This paper	N/A
VEGFA_5’ UTR UAU_ Forward GTCTGCGCATACCGCCGCCTCACC	This paper	N/A
VEGFA_5’ UTR UAU_ Reverse GGTGAGGCGGCGGTATGCGCAGAC	This paper	N/A
VEGFA_5’ UTR UGC_ Forward CACTGTCTGCGCGCACCGCCGCCTC	This paper	N/A
VEGFA_5’ UTR UGC_ Reverse GAGGCGGCGGTGCGCGCAGACAGTG	This paper	N/A
VEGFA_5’ UTR AAA_ Forward GAGCACTGTCTGCGCTTTCCGCCGCCTCACCCG	This paper	N/A

VEGFA_5' UTR AAA_ Reverse CGGGTGAGGCGGCGGAAAGCGCAGACA GTGCTC	This paper	N/A
VEGFA_5'UTR Ins_Forward TGTCTGCGCACAAAACCGCCGCCTCA	This paper	N/A
VEGFA_5'UTR Ins_Reverse GTGAGGCGGCGGTTTTGTGCGCAGACA	This paper	N/A
VEGFA_5'UTR Del_Forward TGTCTGCGCACACCGCCTCACCCG	This paper	N/A
VEGFA_5'UTR Del_Reverse CGGGTGAGGCGGTGTGCGCAGACA	This paper	N/A
ANG_5' UTR Forward GGG GTA CCG TCC TGC CAA AGA AAG CAG C	This paper	N/A
ANG_5' UTR Reverse GCT CTA GAC TCT TCC AAC ACA GGC TCC T	This paper	N/A
hTRS qRT-PCR Forward GTAAGCCATGATGGTGA	This paper	N/A
hTRS qRT-PCR Reverse CTGCCTGTTTGCTGCGG	This paper	N/A
h4EHP qRT-PCR Forward CAGCACACAGAAAGATGGTGA	This paper	N/A
h4EHP qRT-PCR Reverse CTCCAGAACTGCTCCACAGAG	This paper	N/A
hVEGFA qRT-PCR Forward GCACCCATGGCAGAAGG	This paper	N/A
hVEGFA qRT-PCR Reverse CTCGATTGGATGGCAGTAGCT	This paper	N/A
hGAPDH qRT-PCR Forward AGCCACATCGCTCAGACAC	This paper	N/A
hGAPDH qRT-PCR Reverse GCCCAATACGACCAAATCC	This paper	N/A
hANG qRT-PCR Forward AGAAGCGGGTGAGAAACAA	This paper	N/A
hANG qRT-PCR Reverse TGTGGCTCGGTACTGGCATG	This paper	N/A
mTRS qRT-PCR Forward GCTCTAGATTATTCTGCCAGAATGGAATC ATGTTC	This paper	N/A
mTRS qRT-PCR Reverse ATAAGAATGCGGCCGCTAAAGGCAGAAA AAGATAGC	This paper	N/A
m4EHP qRT-PCR Forward GATGGAGGTCGAGCTGAGTTGAATAAGG CAGAAAAAGA	This paper	N/A
m4EHP qRT-PCR Reverse TCTTTTTCTGCCTTATTCAACTCAGCTCGA CCTCCATC	This paper	N/A
mVEGFA qRT-PCR Forward ATAGCGGATGGAAAACCCTGC	This paper	N/A
mVEGFA qRT-PCR Reverse TATCGCCTACCTTTTGGGACG	This paper	N/A
mGAPDH qRT-PCR Forward AGGCCGGTGCTGAGTATGTC	This paper	N/A
mGAPDH qRT-PCR Reverse TGCCTGCTTCACCACCTTCT	This paper	N/A

Morpholino: *trs* MO: GTGATTCTTCAAAACTGACCTCCCA	Gene-Tools	N/A
Morpholino: *4ehp* MO: GCGTGTGTGTAGGTTACCGAAGCA	Gene-Tools	N/A
Morpholino: standard control MO: CCTCTTACCTCAGTTACAATTTATA	Gene-Tools	http://www.gene-tools.com/custom_morpholinos_controls_endmodifications#standardcontrols
**Recombinant DNA**		
pIRES-FLAG	Gack et al., 2008	N/A
eIF4E1-FLAG	This paper	N/A
eIF4E2-FLAG	This paper	N/A
eIF4A-FLAG	This paper	N/A
eIF4G-FLAG	This paper	N/A
PABP-FLAG	This paper	N/A
eIF3C-FLAG	This paper	N/A
eIF3D-FLAG	This paper	N/A
eIF3E-FLAG	This paper	N/A
eIF3F-FLAG	This paper	N/A
eIF3G-FLAG	This paper	N/A
eIF3H-FLAG	This paper	N/A
eIF3I-FLAG	This paper	N/A
eIF3J-FLAG	This paper	N/A
eIF3K-FLAG	This paper	N/A
eIF3L-FLAG	This paper	N/A
eIF3M-FLAG	This paper	N/A
pCMV6-eIF4E3-Myc-FLAG	OriGene	Cat#RC225275
pCMV6-eIF3A-Myc-FLAG	OriGene	Cat#RC221766
pCMV6-eIF3B-Myc-FLAG	OriGene	Cat#RC200612
pEXPR-IBA103	IBA	Cat#2-3503-000
TRS-Strep	This paper	N/A
TRS M60K-Strep	This paper	N/A
TRS R663L/E680L/R689L-Strep	This paper	N/A
AlaRS-Strep	This paper	N/A
CRS-Strep	This paper	N/A
FRS-Strep	This paper	N/A
HRS-Strep	This paper	N/A
IRS-Strep	This paper	N/A
KRS-Strep	This paper	N/A
NRS-Strep	This paper	N/A
QRS-Strep	This paper	N/A
RRS-Strep	This paper	N/A
SRS-Strep	This paper	N/A
WRS-Strep	This paper	N/A
YRS-Strep	This paper	N/A
pBiFC-VN173	Addgene	Cat#22010
FLAG-AlaRS-VN	This paper	N/A
FLAG-EPRS-VN	This paper	N/A
FLAG-KRS-VN	This paper	N/A
FLAG-TRS-VN	This paper	N/A
FLAG-WRS-VN	This paper	N/A

pBiFC-VC155	Addgene	Cat#22011
HA-4EHP-VC	This paper	N/A
HA-eIF4E1-VC	This paper	N/A
HA-eIF4E3-VC	This paper	N/A
pEBG-GST	Addgene	Cat#22227
GST-TRS Full	This paper	N/A
GST-TRS UNE-T	This paper	N/A
GST-TRS TGS/ED	This paper	N/A
GST-TRS CD	This paper	N/A
GST-TRS ABD	This paper	N/A
pMBP-parallel1	Sheffield et al., 1999	N/A
pMBP-His-4EHP$_{45-234}$-TRS$_{30-74}$	This paper	N/A
pcDNA3.1/Myc-His A	Invitrogen	Cat#V800-20
Myc-hTRS	This paper	N/A
Myc-hTRS Y53F	This paper	N/A
Myc-hTRS Y55F	This paper	N/A
Myc-hTRS M60K	This paper	N/A
Myc-hTRS C413S	This paper	N/A
Myc-mTRS	This paper	N/A
Myc-zTRS	This paper	N/A
Myc-zTRS D50I	This paper	N/A
Myc-zTRS I55D	This paper	N/A
Myc-nTRS	This paper	N/A
Myc-yTRS	This paper	N/A
pcDNA3.1(+)	Invitrogen	Cat#V790-20
HA-h4EHP	This paper	N/A
HA-h4EHP H54N	This paper	N/A
HA-h4EHP F94W/L156K	This paper	N/A
HA-h4EHP H54N/F94W/L156K	This paper	N/A
HA-m4EHP	This paper	N/A
HA-z4EHP	This paper	N/A
HA-n4EHP	This paper	N/A
pCMV-HA	Clontech	Cat#631604
HA-y4EHP	This paper	N/A
pET22b(+)	Novagen	Cat#69744-3
hTRS-6xHis	This paper	N/A
hTRS M60K-6xHis	This paper	N/A
hTRS C413S-6xHis	This paper	N/A
hTRS$_{1-74}$-6xHis	This paper	N/A
zTRS$_{1-69}$-6xHis	This paper	N/A
fTRS$_{1-50}$-6xHis	This paper	N/A
pGST-Parallel1	Sheffield et al., 1999	N/A
GST-hTRS	This paper	N/A
GST-h4EHP$_{45-234}$	This paper	N/A
GST-h4EHP$_{45-245}$	This paper	N/A
GST-h4EHP	This paper	N/A
GST-z4EHP	This paper	N/A
GST-f4EHP	This paper	N/A
pHis-GST-parallel1	pGST-parallel1 modified	N/A
His-GST-eIF4A	This paper	N/A
His-GST-PABP	This paper	N/A

pAc5.1/V5-His	Invitrogen	Cat#V411020
fTRS-V5	This paper	N/A
hTRS-V5	This paper	N/A
pAc5.1B-lambdaN-HA	Addgene	Cat#21302
HA-f4EHP	This paper	N/A
HA-h4EHP	This paper	N/A
pGL2-Basic	Promega	Cat#E1641
pGL2-5'UTR-167	This paper	N/A
pGL2-5'UTR-749	This paper	N/A
pGL2-5'UTR-167 CGU	This paper	N/A
pGL2-5'UTR-167 UAU	This paper	N/A
pGL2-5'UTR-167 UGC	This paper	N/A
pGL2-5'UTR-167 AAA	This paper	N/A
pGL2-5'UTR-167 Δ18bp_up	This paper	N/A
pGL2-5'UTR-167 Δ18bp_down	This paper	N/A
pGL2-5'UTR-167 Ins	This paper	N/A
pGL2-5'UTR-167 Del	This paper	N/A
pGL2-5'UTR-553 ANG	This paper	N/A
pRL-SV40	Promega	Cat#E2231
pCS2+-mCherryRed	This paper	N/A
pCS2+-zTRS WT	This paper	N/A
pCS2+-zTRS I55D	This paper	N/A
pCS2+-z4EHP WT	This paper	N/A
**Other**		
MagStrep "type3" XT beads	IBA	Cat#2-4090-002
rProtein G Agarose	Invitrogen	Cat#15920-010
Protein A/G Plus-Agarose	Santa Cruz Biotechnology	Cat#sc-2003
Ni-NTA Agarose	QIAGEN	Cat#30230
Anti-FLAG M2 Magnetic Beads	Sigma-Aldrich	Cat#M8823
C-Myc Agarose Affinity Gel	Sigma-Aldrich	Cat#A7470
Superdex75, 16/60 column	GE Healthcare	Cat#17-1068-01
RNeasy mini kit	QIAGEN	Cat#74106
Threonine, L-[3-3H]	ARC	Cat#ART0330
L-[^{35}S]-Methionine	PerkinElmer	Cat#NEG709A
tRNA from Baker's Yeast	Roche	Cat#10109495001
Immobilized 2'/3'-EDA-m7GTP	Jena Bioscience	Cat#AC-142S
7-Methyl-GTP, Sepharose 4B	GE Healthcare	Cat#27-5025-01
Glutathione Sepharose 4B	GE Healthcare	Cat#17-0756-01
Strep-Tactin Superflow column	IBA	2-1207-001
Amicon Ultra-4 centrifugal unit	Millipore	Cat#801024
iTaq Universal SYBR Green Supermix	Bio-Rad	Cat#172-5120
Matrigel Matrix Growth Factor Reduced	BD Bioscience	Cat#356230
24-well Transwell chambers	Corning	Cat#3422
Homogenizer Microtube set	COSMOBIO	Cat#ISO-228AM
