# Supplementary material for: A threonyl-tRNA synthetase-mediated translation initiation machinery
Source: Nat Commun. 2019 Mar 22;10:1357. doi: 10.1038/s41467-019-09086-0 (PMC6430810; doi:10.1038/s41467-019-09086-0)
Supplement: Supplementary file 3 — Reporting summary [file 41467_2019_9086_MOESM3_ESM.pdf]

nature research

Corresponding author(s): NCOMMS-18-08008C

Last updated by author(s): Jan 27, 2019

Reporting Summary

Nature Research wishes to improve the reproducibility of the work that we publish. This form provides structure for consistency and transparency in reporting. For further information on Nature Research policies, see Authors & Referees and the Editorial Policy Checklist.

Statistics

For all statistical analyses, confirm that the following items are present in the figure legend, table legend, main text, or Methods section.

n/a	Confirmed	
☐	☒	The exact sample size (*n*) for each experimental group/condition, given as a discrete number and unit of measurement
☐	☒	A statement on whether measurements were taken from distinct samples or whether the same sample was measured repeatedly
☐	☒	The statistical test(s) used AND whether they are one- or two-sided<br>*Only common tests should be described solely by name; describe more complex techniques in the Methods section.*
☒	☐	A description of all covariates tested
☒	☐	A description of any assumptions or corrections, such as tests of normality and adjustment for multiple comparisons
☐	☒	A full description of the statistical parameters including central tendency (e.g. means) or other basic estimates (e.g. regression coefficient) AND variation (e.g. standard deviation) or associated estimates of uncertainty (e.g. confidence intervals)
☒	☐	For null hypothesis testing, the test statistic (e.g. *F*, *t*, *r*) with confidence intervals, effect sizes, degrees of freedom and *P* value noted<br>*Give P values as exact values whenever suitable.*
☒	☐	For Bayesian analysis, information on the choice of priors and Markov chain Monte Carlo settings
☒	☐	For hierarchical and complex designs, identification of the appropriate level for tests and full reporting of outcomes
☒	☐	Estimates of effect sizes (e.g. Cohen's *d*, Pearson's *r*), indicating how they were calculated

Our web collection on statistics for biologists contains articles on many of the points above.

Software and code

Policy information about availability of computer code

Data collection	RIP-seq libraries were sequenced on Hiseq-2000 sequencer (Illumina) using 101-bp paired-end reads.
Data analysis	We used the following software: Bowtie2 (v.2.1.0), COOT, Cufflinks (v.2.2.1), DAVID, HKL2000 package, ImageJ, Mev (v.4.9.0), Origin program (v. 7.0), PHENIX, Prism 6, PYTHON (v.2.7.6), R (v.3.1.0), and Tophat2 (v.2.0).

For manuscripts utilizing custom algorithms or software that are central to the research but not yet described in published literature, software must be made available to editors/reviewers. We strongly encourage code deposition in a community repository (e.g. GitHub). See the Nature Research guidelines for submitting code & software for further information.

Data

Policy information about availability of data

All manuscripts must include a data availability statement. This statement should provide the following information, where applicable:

- Accession codes, unique identifiers, or web links for publicly available datasets
- A list of figures that have associated raw data
- A description of any restrictions on data availability

Coordinates and other structure-related information have been deposited in the PDB under PDB code 5XLN, RIP-seq data have been deposited in the GEO data base under accession code GSE120182. All data supporting the findings of this study are available within the article and its supplementary information files, or from the corresponding author upon request.

Field-specific reporting

Please select the one below that is the best fit for your research. If you are not sure, read the appropriate sections before making your selection.

☒ Life sciences ☐ Behavioural & social sciences ☐ Ecological, evolutionary & environmental sciences

For a reference copy of the document with all sections, see nature.com/documents/nr-reporting-summary-flat.pdf

Life sciences study design

All studies must disclose on these points even when the disclosure is negative.

Sample size	Morphologically similar individual embryos (8 ~10 embryos per conditions) were randomly selected for imaging and counting. Exact numbers of embryos analyzed are included in the bar graph in Fig. 5f, 5h, and in Supplementary Fig. 7d.
Data exclusions	Embryos that were obviously delayed due to the injection process, based on the length of the body, were excluded for the further analysis.
Replication	Experiments were done to verify reproducibility of the experimental finding at least three times. Attempts at replication were successful.
Randomization	A method of randomization was not used except for animal studies.<br>Morphologically similar individual embryos (8 ~10 embryos per conditions) were randomly selected for imaging and counting.
Blinding	No blinding process was performed for the analysis but we strived to exclude any bias by randomly selecting the order of imaging and/or measurements.

Reporting for specific materials, systems and methods

We require information from authors about some types of materials, experimental systems and methods used in many studies. Here, indicate whether each material, system or method listed is relevant to your study. If you are not sure if a list item applies to your research, read the appropriate section before selecting a response.

Materials & experimental systems

n/a	Involved in the study	
☐	☒	Antibodies
☐	☒	Eukaryotic cell lines
☒	☐	Palaeontology
☐	☒	Animals and other organisms
☒	☐	Human research participants
☒	☐	Clinical data

Methods

n/a	Involved in the study	
☒	☐	ChIP-seq
☒	☐	Flow cytometry
☒	☐	MRI-based neuroimaging

Antibodies

Antibodies used	Details about all antibodies used in this study are provided in the Supplementary Table 2.
Validation	Validation is provided on the manufacturers websites for each product and can be seen in the representative gating in the manuscript.

Eukaryotic cell lines

Policy information about cell lines

Cell line source(s)	Details about all cell lines used in this study are provided in the Supplementary Table 2.
Authentication	Cell lines were obtained from original sources and were not further authenticated.
Mycoplasma contamination	Cell lines were free of mycoplasma.
Commonly misidentified lines<br>(See ICLAC register)	N/A

Animals and other organisms

Policy information about studies involving animals; ARRIVE guidelines recommended for reporting animal research

Laboratory animals	Details about all laboratory animals used in this study are provided in the Supplementary Table 2.
Wild animals	This study did not involve wild animals.
Field-collected samples	This study did not involve field-collected samples.
Ethics oversight	Zebrafish husbandry and animal care were carried out in accordance with guidelines of KRIBB and experimental protocols were approved by KRIBB-IACUC (approval number: KRIBB-AEC-17117).

Note that full information on the approval of the study protocol must also be provided in the manuscript.
